# Hand/foot splitting and the ‘re-evolution’ of mesopodial skeletal elements during the evolution and radiation of chameleons

**DOI:** 10.1186/s12862-015-0464-4

**Published:** 2015-09-18

**Authors:** Raul E. Diaz, Paul A. Trainor

**Affiliations:** Department of Biology, La Sierra University, Riverside, CA 92515 USA; Natural History Museum of Los Angeles County, Los Angeles, CA 90007 USA; Stowers Institute for Medical Research, Kansas City, MO 64110 USA; Department of Anatomy and Cell Biology, University of Kansas Medical Center, Kansas City, KS 66160 USA

## Abstract

**Background:**

One of the most distinctive traits found within Chamaeleonidae is their split/cleft autopodia and the simplified and divergent morphology of the mesopodial skeleton. These anatomical characteristics have facilitated the adaptive radiation of chameleons to arboreal niches. To better understand the homology of chameleon carpal and tarsal elements, the process of syndactyly, cleft formation, and how modification of the mesopodial skeleton has played a role in the evolution and diversification of chameleons, we have studied the Veiled Chameleon (*Chamaeleo calyptratus*). We analysed limb patterning and morphogenesis through *in situ* hybridization, *in vitro* whole embryo culture and pharmacological perturbation, scoring for apoptosis, clefting, and skeletogenesis. Furthermore, we framed our data within a phylogenetic context by performing comparative skeletal analyses in 8 of the 12 currently recognized genera of extant chameleons.

**Results:**

Our study uncovered a previously underappreciated degree of mesopodial skeletal diversity in chameleons. Phylogenetically derived chameleons exhibit a ‘typical’ outgroup complement of mesopodial elements (with the exception of centralia), with twice the number of currently recognized carpal and tarsal elements considered for this clade. In contrast to avians and rodents, mesenchymal clefting in chameleons commences in spite of the maintenance of a robust apical ectodermal ridge (AER). Furthermore, *Bmp* signaling appears to be important for cleft initiation but not for maintenance of apoptosis. Interdigital cell death therefore may be an ancestral characteristic of the autopodium, however syndactyly is an evolutionary novelty. In addition, we find that the pisiform segments from the ulnare and that chameleons lack an astragalus-calcaneum complex typical of amniotes and have evolved an ankle architecture convergent with amphibians in phylogenetically higher chameleons.

**Conclusion:**

Our data underscores the importance of comparative and phylogenetic approaches when studying development. Body size may have played a role in the characteristic mesopodial skeletal architecture of chameleons by constraining deployment of the skeletogenic program in the smaller and earliest diverged and basal taxa. Our study challenges the ‘re-evolution’ of osteological features by showing that ‘re-evolving’ a ‘lost’ feature *de novo* (contrary to Dollo’s Law) may instead be due to so called ‘missing structures’ being present but underdeveloped and/or fused to other adjacent elements (cryptic features) whose independence may be re-established under changes in adaptive selective pressure.

**Electronic supplementary material:**

The online version of this article (doi:10.1186/s12862-015-0464-4) contains supplementary material, which is available to authorized users.

## Background

Understanding the complex relationship between genotype and phenotype requires an integrative and interdisciplinary biological framework [1]. Loci known to be involved in development, morphogenesis and in the pathogenesis of congenital malformations have been identified through forward genetic approaches, genomic mapping or genome wide association studies [2–4]. Concurrent with studying how malformations arise, cellular and genetic mechanisms have been uncovered which have direct consequences on our understanding of the processes governing normal development [5, 6]. Examining the development of innovations and novelties [7] in natural groups provides an understanding of how body plans have been molded by natural selection [8] as optimal phenotypes through adaptive evolution.

**Fig. 1:**
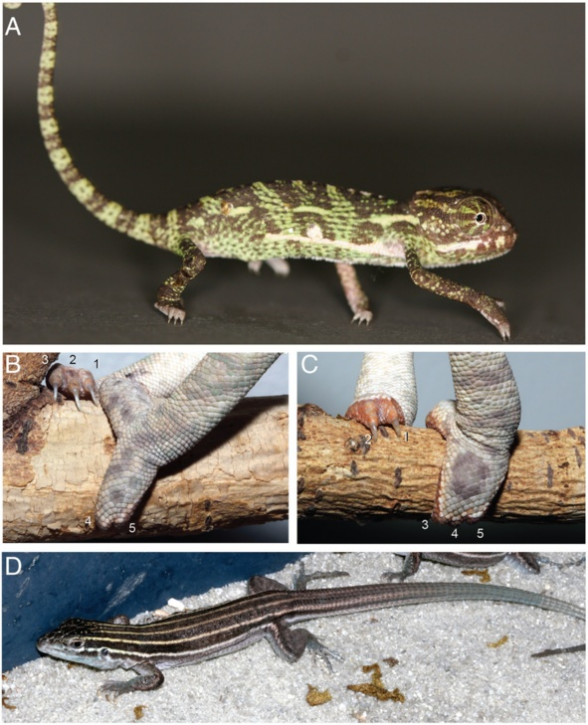
Chameleon autopodia. **a** The Veiled Chameleon (*Chamaeleo calyptratus*) has a laterally compressed body, prehensile tail, turreted and independent eyes, a projectile tongue and zygodactylous cleft autopdia. **b** Chameleon hand digits I, II, III are syndactylous medially with digits IV, V are bound laterally. Feet (**c**) have digits I, II syndactylous medially while digits III, IV, V are bound laterally. **d** The Desert Grassland Whiptail Lizard (*Aspidoscelis uniparens*) was used as for comparison due to its ‘typical’ terrestrial lizard body plan

Limbs and digits were key innovations in the evolution and diversification of tetrapods [7, 9–11]. While much has been learned about gene function during morphogenesis and differentiation of the tetrapod limb [12], most studies examining non-traditional model organisms have focused on limb reduction or loss of distal elements of the autopodium [13–19]. Within Reptilia, the Order Squamata (lizards, snakes and amphisbaenians) comprises more than 53 lineages representing independent limb reduction toward a snake-like body form [20]. However, one of the most dramatic limb skeletal modifications is found in the hands and feet of the family Chamaeleonidae, the chameleons [21–23]. Chamaeleonidae limbs have been modified from the generalized terrestrial tetrapod plan to exhibit an architecture highly adapted for an arboreal lifestyle. This was facilitated through evolution of a midline autopodial cleft (ectrodactyly; Fig. 1a) and two opposable syndactylous bundles (different finger clusters between hands and feet that retain interdigital tissue) of digits that are highly mobile (zygodactyly; Fig. 1b and c). In addition, the proximal skeleton of the autopodium (the wrist and ankle, or mesopodia) was modified through a reduction in the number of bone elements. Furthermore, those elements that remain are enlarged and form a ball-and-socket joint between the autopodium and forearm (zeugopodium). This specialized joint allows for greater rotation of the wrist and ankle, which is important while climbing. Limb modifications such as these facilitated an increase in precision, security and mobility in the three-dimensional chameleon arboreal environment. While other lizards are also arboreal (i.e., geckos, anoles), chameleons differ by using zygodactyly and gripping of branches rather than depending on claws and epidermal modifications (Fig. 1d).

Few studies to date have focused on the development of chameleon autopodial architecture [24, 25], leaving unanswered the question of how the highly conserved tetrapod pentadactyl autopodium [26] was modified during chameleon evolution. To address this issue, we have developed and utilized the Veiled Chameleon (*Chamaeleo calyptratus*), which offers key advantages over other lizard and chameleon species [27, 28]. One of the most important and distinctive developmental traits in *C. calyptratus* is their slow rate of embryogenesis (200 days for eggs incubated at 26 **°**C), which facilitates the examination of fine, progressive, morphological changes [29].

Chameleons provide an important example of how developmental mechanisms may be modified through heterochrony and constrained by body size to produce highly adaptive morphologies. With respect to the limb, a proximal reduction in the number of carpal and tarsal elements in adult chameleons with concurrent distal cleft formation of autopodia have facilitated the evolution of complex zygodactylous grasping in a three dimensional arboreal environment. We collected *C. calyptratus* embryos at various time points to study gene expression, morphogenesis, and skeletal and histological development of autopodial elements. We also examined 8 of the currently recognized 12 genera of chameleons and 2 outgroup taxa to understand the evolution and diversity of carpal and tarsal elements across this phenotypically derived clade of arboreal lizards. A reversal to an increased number of carpal and tarsal elements together with increased independence of elements, appears to have played a significant role in the evolution of ‘true chameleons’. Such a mechanism could provide more components by which to increase wrist flexion, a biomechanical advantage in a complex arboreal environment.

## Methods

### Embryos

Veiled chameleon eggs were collected at oviposition in the Stowers Institute for Medical Research’s Reptiles and Aquatics Facility and at the La Sierra University Animal Facility. Eggs were subsequently incubated at a constant 26 °C in deli cups with moist vermiculite. Embryos were harvested in cold 1× Phosphate Buffered Saline (PBS) at varying stages, fixed overnight in DEPC-treated 4 % Paraformaldehyde in 1xPBS at 4 °C and later dehydrated to absolute Methanol before storing at −20 °C [27, 28].

### DAPI Helicon imaging (pseudo-SEM)

Whole embryos were collected in cold 1x Phosphate Buffered Saline (PBS) and fixed overnight at room temperature with 1 μl DAPI, dilactate (Sigma®, D9564) (2 mg/ml diluted in DI H_2_O) per 1 ml of PBS on a roller (1 h may be sufficient for small embryos) [30]. Embryos were placed flat in a 50 ml petri dish filled with PBS and imaged on a Leica MZ FLIII with a Carl Zeiss color AxioCam HRc using AxioCam software. The UV filter was used to visualize DAPI fluorescence (Blue). Settings were changed to black and white in the software to generate a grayscale image of the embryo. Z-stack images were taken and subsequently processed with Helicon Focus 5.3 software (Helicon Soft Ltd.), which functions to create a projection of all images into a single high focus photo. This process is analogous to doing a DAPI (grayscale) Z-stack on a confocal, and is useful for generating high resolution images of embryos too large for visualization on a confocal microscope. This method also overcomes some of the limitations of SEM imaging in that the embryos never have to be dehydrated, can be kept at room temperature in fixative indefinitely, can be used on museum specimens, is non-destructive (unlike SEM), and also provides simultaneous tissue resolution below the differentiating skin. A 50 ml plastic petri dish was half-filled with 1 % Agarose and left to solidify. Troughs in the agarose were created to accommodate embryos in various orientations and provide stability during imaging (while filled with PBS or water).

### RNA collection, cDNA synthesis, probe development and in situ hybridization

Embryos between 90 and 104 dpo (days post oviposition; incubated at 26 °C) were collected in cold DEPC-treated 1xPBS. Limbs were pooled together and total RNA was extracted with the RNEasy Mini Kit (Qiagen). Complementary DNA (cDNA) was derived from messenger RNA using the SuperScript® First-Strand Synthesis System for RT-PCR (Life Technologies). Degenerate primers were designed for genes of interest. GoTaq® Colorless MasterMix (Promega) was used for PCR amplification. Reaction products were run on a 1 % Agarose gel and bands of expected sizes were excised, purified using the QiaEx II Gel Extraction Kit (Qiagen), and stored at −20 °C. Amplified fragments were ligated into pGEM®-T Easy Vectors (Promega). DNA was sequenced using Sp6 and T7 primers, and checked *via* BLAST (NCBI) to determine the direction of the insert. Plasmids were digested using the appropriate restriction enzymes for the development of sense and antisense *in situ* hybridization probes using the DIG RNA labeling kit (Sp6/T7) (Roche). Degenerate primers used include: For Sonic Hedgehog (~510 bp) ShhF: 5’-CCGGCTTCGACTGGGTNTAYTA-3’, ShhR: 5’-CATGGGCGGTCAGTGGNGCRTANGC-3’. For Fibroblast Growth Factor 8 (~403 bp) Fgf8F: 5’-CTMRTSCGSACCTACCARCT-3’, Fgf8R: 5’-GGSARSCKCTTCATGAAGTG-3’. For Gremlin 1 (~329 bp) Grem1F: 5’-CAAGGMTCAGCMCAATGAYTC-3’, Grem1R: 5’-YTTCTTGGGCTTGCAGAAG-3’. GenBank accession numbers for the above amplified fragments are [GenBank: KR081935-KR081937].

For *in situ* hybridization, embryos were collected in cold DEPC-treated 1x PBS, and fixed in 4 % paraformaldehyde overnight at 4 °C on a rocker. Specimens were then dehydrated through a graded series of Methanol/PBS-DEPC into 100 % Methanol and stored at −20 °C. Whole mount *in situ* hybridization was performed as previously described [31].

### Micro-Computed Tomography (microCT)

Comparative limb osteological data was obtained by isolating limbs from adult specimens followed by scanning with a Scanco vivaCT40 micro-computed tomography scanner. Dicom data was visualized using Amira 5.0 (FEI).

### Scanning electron microscopy

Embryos were fixed overnight in 2.5 % gluteraldehyde/2 % paraformaldehyde in 1xPBS, rinsed in water (3 × 1 h), incubated in 2 % Osmium Tetroxide (1 h), rinsed again in water (3×1 h), and dehydrated through a graded series of ethanol (30 %, 50 %, 70 %, 80 %, 90 %, and 3×100 %) for 20 min each. Critical point drying was conducted in Tousimis Samdri-795 critical point dryer. Scanning was accomplished on a Hitachi TM-1000 Tabletop Microscope.

### Alcian/Alizarin skeletal preparations

Staining of bone with Alizarin Red (Sigma®, A5533) and cartilage with Alcian Blue 8GX (Sigma®, A-5268) was adapted from Walker and Kimmel [32]. Initially, embryos were fixed in absolute ethanol (EtOH) and dehydrated/stored in absolute EtOH (with late gestation embryos and post-hatching individuals being skinned and eviscerated to allow for staining and clearing). Specimens were stained overnight in alizarin red/alcian blue solution at room temperature with rocking. On the following day, specimens were rinsed in water, and, depending on the size of the specimen, were incubated in 4 %, 2 %, 1 % or 0.5 % KOH until sufficient soft tissue digestion had occurred. Specimens were then cleared in 25 % glycerol in 0.5 % KOH, followed by 50 % glycerol in 0.5 % KOH before being post-fixed in 2 % formaldehyde in 50 % Glycerol/1xPBS, which is also the permanent storage media for specimens. Adult museum specimen catalogue numbers (California Academy of Sciences) are included in the images in Additional file 1: Figure S1, with each species represented by its unique tiff file labeled A-N. Specimen measurements can be found in Additional file 7.

### H&E paraffin histology

Embryos were fixed in 4 % PFA overnight, dehydrated through an ethanol series, cleared in Xylene, and embedded in paraffin. 8–10 μm sections cut onto slides were deparaffinized with xylene, re-hydrated through an ethanol series into water and counterstained with hematoxylin and eosin.

### Whole embryo culture

Eggs were incubated at 26 °C for 100 days and candled to check for viable embryos. Healthy eggs limit light passing through the egg (Additional file 4: Figure S4A) and also exhibit membrane vasculature, whereas infertile or unhealthy eggs appear quite clear (Additional file 4: Figure S4B). Pin-hole punctures with forcep tips allowed for very clear and viscous albumin/chorioallantoic fluid to leak onto the egg shell, while infertile eggs burst, releasing a creamy white yolk (Additional file 4: Figure S4C). Shell opening ruptures the chorioallantoic membrane (Additional file 4: Figure S4D), however the embryo remains within the amnion embedded within the yolk sac. The amnion can be seen as a remnant of membranes externally (bottom right of image). The supine position of the embryo is associated with the constraint imposed by the umbilical arteries and veins leading toward this thin transparent membrane where the yolk sac and allantoic membranes both diverge (Additional file 4: Figure S4E). Care must be taken to cut through the clear membrane without severing the umbilical veins/arteries. The egg yolk was cut to form a ‘blanket’ below the embryo while retaining the remaining chorioallantoic membrane and yolk sac vasculature (Additional file 4: Figure S4E-G). Embryos were then placed in organ culture media within a 6-well plate (H; with sufficient media to give buoyancy to embryos). Embryos were cultured (with plate lids on) in an Exo-Terra Thermoelectric Reptile Incubator (I; Model PT2499) for 4 days at 26 °C under high humidity. Media was changed daily.

### Media for culture

Culture media consisted of 1.5 ml of Fetal Bovine Serum (heat inactivated; Sigma F4135), 8.3 ml DMEM/F-12 50/50 with L-glutamine (Corning 15-090-CV), 10 μl of 50 mg/ml Ascorbic Acid for final 50 μm/ml concentration (Fisher #S25184). 200 μl of Pen-Strep (for total of 2 %; MP Biomedicals 1670049) was added to limit bacterial contamination.

### Nile blue staining

Embryos were incubated in 1:1000 dilution of a 1.5 % stock solution of Nile Blue in culture media for 40 min in a 26 °C incubator, followed by two 30 min washes in PBS with rocking at 4 °C prior to photographing.

### LDN193189 (Dorsomorphin) treatment

Dorsomorphin (Stemgent, Stemolecule LDN193189, 04–0074) was added to a final concentration of 15 μM in culture media (stock solution dissolved in DMSO).

### Animal research

All procedures were formally approved by the Institutional Animal Care and Use Committee at the Stowers Institute for Medical Research and the Animal Care and Use Committee at La Sierra University. No wild caught animals were used in this study. All embryos studied were produced in house.

## Results

### Interdigital clefting is independent of a robust AER

**Fig. 2:**
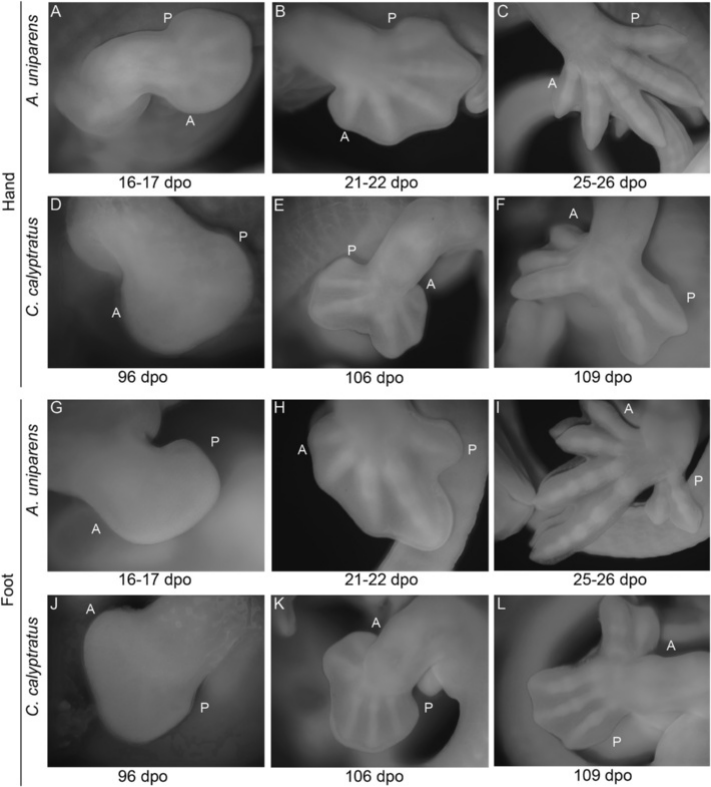
Comparative morphogenesis of lizard autopodia. In *Aspidoscelis uniparens*, the digital plate progresses through a round shape with the pentadactyl digital rays developing within and visible through the thin tissue (**a**). Subsequent thickening of digital rays and thinning of the interdigital mesenchyme (**b**) ultimately leads to loss of the interdigital tissue through apoptosis (**c**). In the chameleon hand, a semi round digital plate develops (**d**) with subsequent distal flattening. Clefting initiates at this distally flattened location (**e**). Two syndactylous digit bundles are approximately 180° apart in the anterior-posterior plane. The *A. uniparens* foot also passes through a semi round digital plate stage (**g**) which subsequently presents digit differentiation with subsequent interdigital cell death (**h**-**i**). The chameleon foot undergoes the same stages as in the hand (**d**-**f**), but terminates with a different complement of digits in syndactyly (**j**-**l**). dpo = days post oviposition; A = Anterior; *P* = Posterior

To begin to understand the mechanisms governing the modified hand and foot morphology of chameleons, we compared autopodial development in the Veiled Chameleon to the Desert Grassland Whiptail Lizard (Teiidae: *Aspidoscelis uniparens*), which serves as a reference species due to its overall ‘typical’ lizard limb and body plan. In lizards, it takes approximately 10 days for limb development to progress from a round digital plate to the typical pentadactyl pattern which coincides with the onset of interdigital cell death (ICD) (Fig. 2a-c, j-l). In contrast, autopodial morphogenesis in *C. calyptratus* lasts approximately 14–21 days from the rounded paddle stage through cleft completion. Phenotypic divergence between these species commences at the late round digital plate stage when distal flattening occurs in the chameleon forelimb and hindlimb (Fig. 2d, j; compare to Fig. 2a and g). Subsequent anteroposterior expansion of the flattened region delimits the clefting domain such that forelimb and hindlimb digits differ in their syndactylous clusters, with digits grouped as (1,2,3) (4,5) (Fig. 2e and f) and (1,2) (3,4,5) (Fig. 2k and l), respectively (compare to Fig. 2b-c, h, i of *A. uniparens*).

**Fig. 3:**
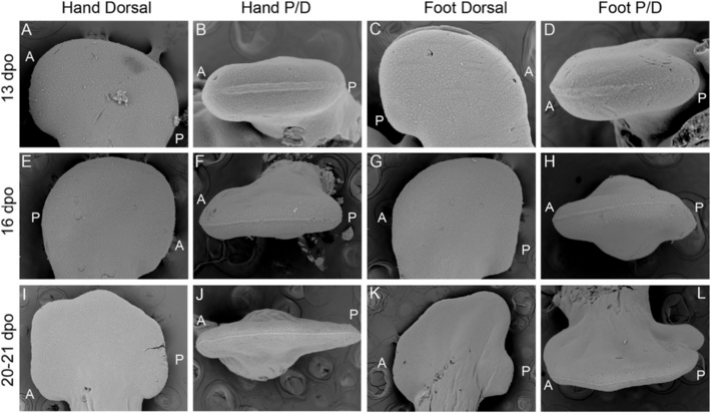
SEM of *Aspidoscelis uniparens* hand and foot morphogenesis. *Aspidoscelis uniparens* hands and feet undergo the same stages of morphogenesis as presented in Fig. 2 in dorsal view (**a**, **e**, **i**; **c**, **g**, **k**), respectively. In distal view (**b**, **f**, **j**), the hand shows an Apical Ectodermal Ridge (AER) which is initially very robust as a distal ectodermal thickening and is present at the midline between the dorsal and ventral half of the developing limb. At later stages, the AER narrows significantly as it spans a greater anterior-posterior domain. In the foot (**d**, **h**, **l**), we see a similar situation with a very straight and stereotypical AER which is initially robust and later thins

**Fig. 4:**
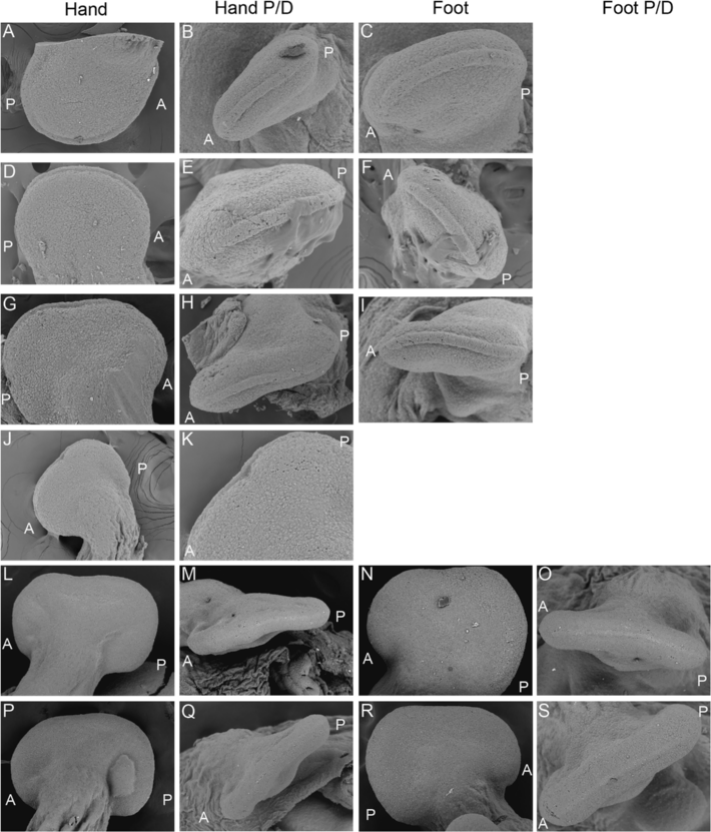
SEM of *Chamaeleo calyptratus* hand and foot morphogenesis. Early stages of morphogenesis show that the chameleon hand develops as a round digital plate (**a**) which subsequently develops a distal flattening (**d**, **g**). In dorsal view, these stages present a very robust AER, relatively larger than that present in *A. uniparens* (Fig. 3) while in distal view the AER is seen as having a greater thickness along the dorsoventral midline (**b**, **e**, **h**). Significantly, during the stage at which the distal autopodium begins to flatten (**g**), the distal AER is no longer the stereotypical A-P flattened ectodermal thickening but is instead arched ventrally (**h**, **i**). Surprisingly, despite having a robust distal AER, proximal mesenchymal cleft formation has already begun (**j**, **k**). At later stages (**l**-**o**, **p**-**s**), both the forelimb and hindlimb expand the cleft while maintaining the AER quite robust until the thickness tapers and narrows at later stages (while the AER also returns to its expected conformation of a straight distal ridge)

Current knowledge of cleft formation in autopodia comes from examination of avian and mammalian systems as well as linkage studies in humans, with all work supporting a failure to maintain the integrity of the Apical Ectodermal Ridge (AER), particularly along the distal midline [33–35]. Loss of the AER inhibits not only distal outgrowth of the limb but also leads to the loss or splitting of the digital rays. To determine whether or not AER destabilization is the primary factor driving cleft formation, we examined the AER of *A. uniparens* in order to first understand how a ‘typical’ AER is structured in pentadactyl lizard autopodia (Fig. 3). At 13 dpo (days post oviposition), a robust distal ectodermal thickening (Fig. 3a-d) is present at the dorso-ventral boundary of the forelimbs and hindlimbs. At 16 dpo (Fig. 3e-h) the AER begins to narrow as the digital rays differentiate within the underlying mesenchyme. By 20–21 dpo (Fig. 3i-l) the AER further narrows prior to the initiation of interdigital cell death. By comparison, in chameleon autopodia, the AER is thicker and more prominent (Fig. 4a-i). Interestingly, upon distal flattening of the digital plate (Fig. 2d, j; Fig. 4g, J, k), commencement of distal clefting in chameleon interdigital tissue is visible despite the retainment of a very robust AER. Syndactylous digit clusters diverge toward the anterior and posterior poles, increasing the angle between clusters and further expanding the cleft. The proximodistal size of the autopodium remains unchanged during these stages. Overall, autopodial shape shifts from a distally flattened rounded paddle to one that is rectangular shaped (Fig. 4l-o). It is interesting to note that as the AER is maintained, it appears slightly thicker in the region overlying the clefting mesenchyme (Fig. 4q, s), which may be compensatory to maintain interdigital tissue proliferation during this midline mesenchymal expansion in concert with digit bundle realignment prior to cleft formation.

#### Autopodial patterning is normal despite a divergent skeleton

**Fig. 5:**
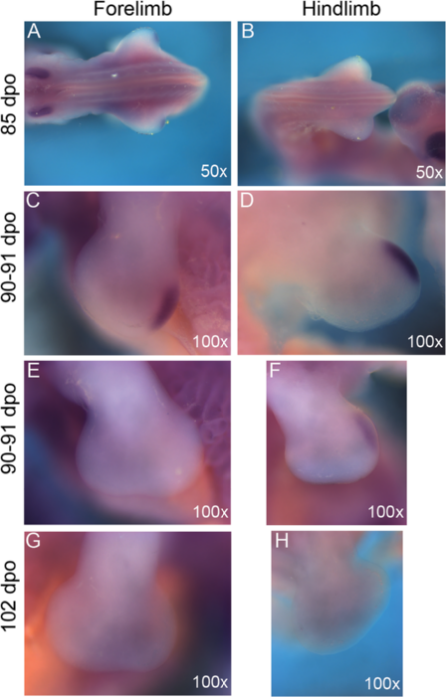
*C. calyptratus* autopodial Sonic Hedgehog (*Shh*) expression. Wholemount *in situ* hybridization indicates that *Shh* is expressed in a similar pattern to other amniotes along the posterior margin of the developing limb within the Zone of Polarizing Activity (ZPA) in the hands (**a**, **c**) and feet (**b**, **d**). Significantly, the expression of *Shh* is lost upon distal flattening of the limb bud in both the hands (**e**, **g**) and feet (**f**, **h**)

Chameleons retain the pentadactyl tetrapod autopodial plan but differ significantly from other tetrapods due to the presence of ectrodactyly and a highly modified mesopodial skeleton. To establish whether early changes in limb patterning influence subsequent development of the novel hand and foot morphology in this group, we characterized the spatiotemporal activity of the major signaling pathways involved in limb development as previously determined in murine and avian species. We initially examined the spatiotemporal activity of Sonic Hedgehog (SHH). *Shh* is known to play a central role in regulating anteroposterior patterning of the digits as well as proliferation of the autopodial mesenchyme which gives rise to the digits. Furthermore, *Shh* plays a feedback role with *Grem1* in the limb mesenchyme and with *Fgf8* in the AER to maintain distal limb outgrowth [12, 36, 37]. To date, *Shh* expression has only been examined during squamate limb development in lizard limbs, and it was shown that the temporal activity of *Shh* expression in the posterior Zone of Polarizing Activity (ZPA) plays a role in determining the number of skeletal elements in the digits of element-reduced species in the scincid genus *Hemiergis*. In this case, shorter expression periods were observed in association with phalangeal and digit reduced taxa [18]. In chameleons, the tetrapod pentadactyl plan is conserved. The phalangeal formulae for the hands and feet of *C. calyptratus* are only slightly modified [hands: 2,3,4,4,3; feet: 2,3,4,4,3; see Fig. 10e-h) relative to the phalangeal formulae of ancestral pentadactyl squamates [hands: 2,3,4,5,3; feet: 2,3,4,5,4] [38, 39]. Therefore, we hypothesized that the spatiotemporal activity of *Shh* would also be conserved in chameleons. *Shh* is in fact expressed in the expected posterior domain demarcating the ZPA in Veiled Chameleons, as it is in the pentadactyl *Hemiergis* [18]. Furthermore, *Shh* is expressed in *C. calyptratus* throughout early limb bud development while the digital plate is rounded (Fig. 5a-d), but is subsequently downregulated upon distal flattening of the digital plates (Fig. 5e-h).

**Fig. 6:**
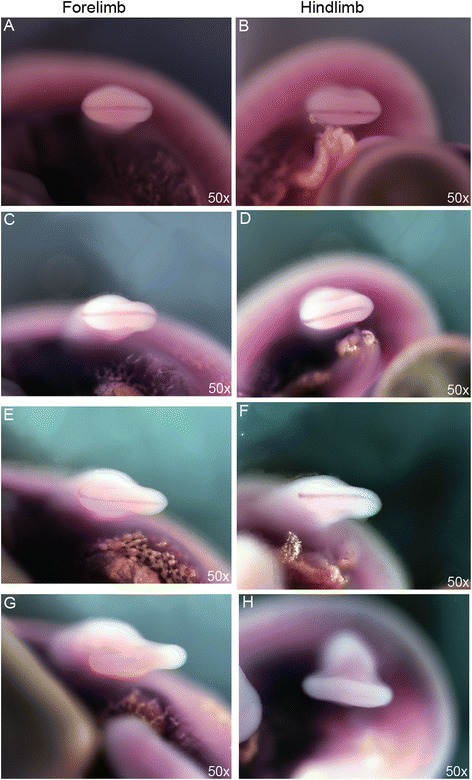
*C. calyptratus* Fibroblast Growth Factor 8 (*Fgf8*) expression in the Apical Ectodermal Ridge. In wholemount *in situ* hybridization, the Apical Ectodermal Ridge expresses *Fgf8* in the highly conserved pattern as seen in tetrapods (**a**-**d**) along the distal anteroposterior boundary of the limb. *Fgf8* expression is maintained in the AER at stages where mesenchymal clefting of the autopodia has already commenced in both the hands and feet, though at a reduced amount prior to complete cleft formation (**e-h**)

The AER forms at the dorsoventral boundary along the distal edge of the limb bud and in mice is the site of expression for members of the Fibroblast Growth Factor family (*Fgf4*, *Fgf9*, *Fgf17*, *Fgf8*) [36, 40]. In particular, *Fgf8* is necessary and sufficient for distal outgrowth and patterning of the growing limb [40]. Temporal removal of the AER at various developmental stages allows for only more proximal elements to form [41]. Using *Fgf8* as a candidate marker for AER integrity (complementing the SEMs in Fig. 4), we find that the AER maintains normal *Fgf8* expression along the anteroposterior distal boundary across all digits as would be expected for a pentadactyl limb (Fig. 6a-h). However, *Fgf8* continues to be expressed in the AER covering the distal boundary of the expanding interdigital domain which will form a cleft during anteroposterior movements of the two syndactylous digit bundles (Fig. 6e-f). Ultimately, *Fgf8* expression diminishes in association with interdigital mesenchymal regression (Fig. 6g-h).

#### Clefting is due to increased cell death

**Fig. 7:**
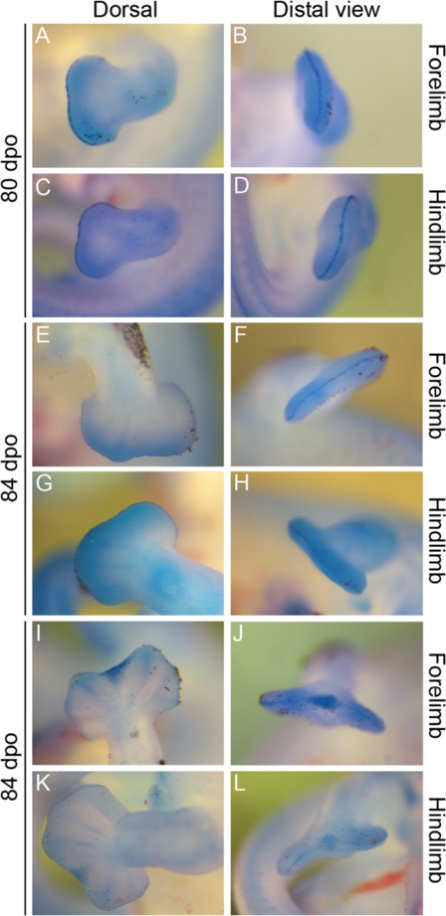
Nile Blue staining for cell death during autopodial morphogenesis. During autopodial morphogenesis, Nile Blue granules are concentrated along the distal periphery of the developing limb within the mesenchyme as well as delimiting the Apical Ectodermal Ridge in both the hands and feet (**a**-**j**). The AER is also highlighted as ventrally arched in distal view (**b**, **d**) and is also clearly dorsoventrally thinner at stages when cleft formation is underway (**h**, **j**). During final stages of cleft morphogenesis, nile blue staining is concentrated within the interdigital mesenchyme (**k**-**n**), with a significantly greater concentration in the interdigital mesenchyme between digits III and IV in the hand (**k**, **l**) and II and III in the foot (**m**, **n**). Reduced Nile Blue is present in the tissue within syndactylous digits. Nile Blue is visible in the AER on the anterior and posterior regions of the limb while absent in the cleft domain in the hand (**k**, **l**)

**Fig. 8:**
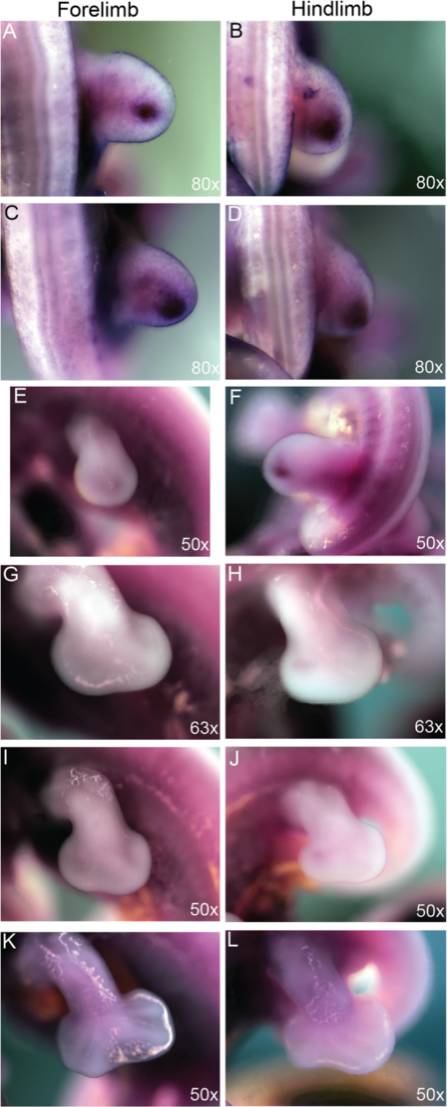
*C. calyptratus* Gremlin 1 (*Grem1*) expression during autopodial morphogenesis. *Grem1* is expressed in the mesenchyme of the autopodium anterior to the ZPA where a feedback loop is known to be present between *Grem1* and SHH, Bmps, and FGF8 (**a**-**f**). *Grem1* appears to decrease its anterior expression during digital plate formation in the forelimb (**e**) and hindlimb (**f**). During distal flattening and cleft formation, *Grem1* does not appear ectopically between syndactylous digits (**g**-**l**)

Recent work exploring digit number reduction [13, 16, 19] supports an important role for cell survival in sculpting adult autopodial elements. The presence of macrophages in interdigital cleft mesenchyme has led to the suggestion that apoptosis is a primary driver of the derived chameleon autopodium [24]. This idea warrants further validation, as mature digit formation generally requires apoptotic removal of interdigital mesenchyme in all known tetrapod limbs [42, 43]. Macrophage associated or non-associated cell death would therefore be expected to be localized to the clefting mesenchyme in most tetrapods, with the exception of taxa with interdigital webbing where cell death is inhibited [44–46]. We used Nile Blue staining as evidence for cell death in whole embryo cultured chameleons at stages during clefting (Fig. 7a-l). While the presence of macrophages is common in the AER to aid its maintenance and extension [47, 48], we also observed increased cell death in the mesenchyme in the distal margin of the autopod. At the onset of interdigital cell death, we observed differential Nile Blue staining between the major clefting domain along the distal midline relative to the interdigital mesenchyme of digits bound in syndactyly (Fig. 7k-n). This reflects the comparative retention of mesenchyme between digits, compared to the cleft. At later stages of embryogenesis, the AER covering the cleft is no longer stained by Nile blue (Fig. 7l, n). In contrast, anterior and posterior syndactylous domains retain Nile blue staining in the AER of the hand (Fig. 7k, l) and foot (Fig. 7m, n), highlighting the requirement for an AER in mesenchymal tissue maintenance. In ducks and bats, *Gremlin1* (*Grem1*), an inhibitor of BMP signaling, is expressed in the interdigital mesenchyme promoting the retention of mesenchymal tissue, resulting in webbing [44]. Chameleons initially exhibit expression of *Grem1* in the developing limb bud mesenchyme anterior to *Shh* in the ZPA [12], which is typical for limbs during outgrowth. However, *Grem1* is subsequently downregulated in interdigital regions where syndactyly occurs (Fig. 8g-l).

Members of the bone morphogenetic protein (Bmp) family play diverse roles in limb morphogenesis. During early limb development BMPs are initially required for formation of the AER [49], but are then negatively regulated by *Grem1* in the limb mesenchyme to allow for activation of Fgfs present in the AER [50–52]. Later during autopodial morphogenesis, *Bmp2, 4,* and *7* are expressed within interdigital mesenchyme prior to and during the process of programmed cell death where soft tissue is removed to delineate each digit [53, 54] (reviewed by [42, 55]). Inhibiting Bmp signaling within interdigital mesenchyme through ectopic expression of Bmp inhibitors such as *Noggin* or *Gremlin* or by dominant negative Bmp Receptors leads to a loss of programmed cell death in chick [46] and mouse [52, 56–58].

**Fig. 9:**
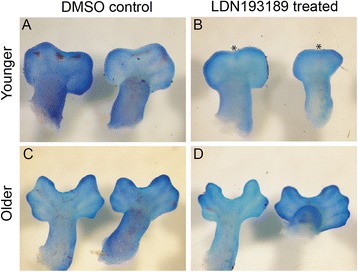
Disruption of Bmp signaling during autopod development. Dorsomorphin (LDN193189) at 15 μM was used to disrupt Bmp signaling during whole embryo *in vitro* culture for 4 days. In the ‘younger’ group of embryos (**a**-**b**) collected prior to distal flattening of the digital plate, treatment led to impaired cleft formation. Asterisks highlight reductions in cleft formation relative to controls. **b** Older embryos collected during the onset of clefting (**c**-**d**) had no effect from the LDN193189 treatment over a 4 day growth period (**d**)

To functionally test the role of active Bmp signaling in establishing the apoptotic program in association with cleft formation in the chameleon limb, we cultured whole embryos *in vitro* in the presence of LDN193189, a pharmacological molecule that inhibits Bmp signaling by blocking Smad activity (Dorsomorphin; [59]). Two groups of embryos were cultured for 4 days. One group termed ‘younger’ were collected at the distally flattened limb paddle stage of development prior to the onset of interdigital cell death (Fig. 9a-b). The ‘older’ group was collected after clefting had commenced (Fig. 9c-d). For ‘older’ embryos cultured at the onset of clefting, tissue loss continued (Fig. 9d) despite exposure to Dorsomorphin (3/3 embryos). However, if Dorsomorphin exposure occured prior to the initiation of cell death, clefting was prevented (Fig. 9b). However, digital ray development was still slightly impaired, and the digits also appeared smaller relative to controls (3/3 embryos). Smaller digital rays in Bmp inhibited mouse limbs are due to direct inhibition of *Sox9* by Fgf, and a delay in chondrification [58]*.* Blocking Bmp activity maintains digital ray cells in an undifferentiated state due to continued Fgf expression in the AER [58]. Treating mouse embryonic limbs with Dorsomorphin during the period of interdigital cell death led to a reduction of Smad phosphorylation in interdigital mesenchyme but did not affect apoptosis [57]. However, treating mouse embryos with Dorsomorphin at earlier stages prior to initiation of apoptosis, did inhibit interdigital cell death. Previous studies genetically perturbing Bmp signaling in chicken and mouse embryos has also led to comparable limb phenotypes [46, 56]. Thus, our study of cleft formation in chameleon embryos reveals that blocking Bmp signaling can inhibit interdigital cell death in a time dependent fashion prior to the onset of programmed cell death.

#### Cryptic diversity of mesopodial skeletal element number in *C. calyptratus* embryos

**Fig. 10:**
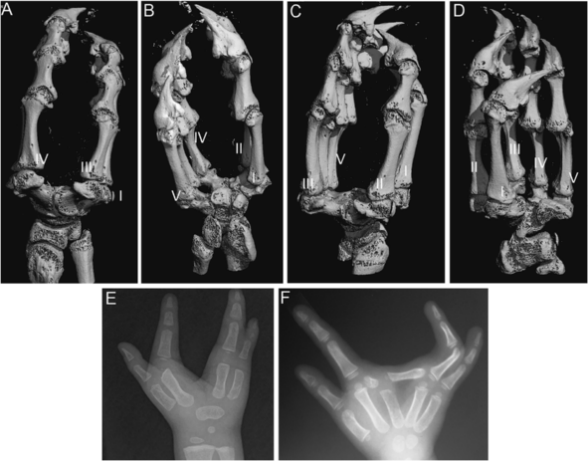
Tetrapod autopodia are dorsoventrally flattened, except in chameleons. Chameleon autopodia (*C. calyptratus*; **a**-**d**) while retaining the pentadactyl complement of digits, not only have a distal cleft separating the digits III and IV in the hand (**a**, **b**) and II and III in the foot (**c**, **d**) but also develop a rosette shaped cluster of metacarpals and metatarsals around a central enlarged distal carpal element. In species (humans) with congenital distal clefting (**e**, **f**), clefting does not alter the dorsoventrally flattened morphology

Limbs and fins come in a variety of shapes and sizes, with variable numbers of skeletal elements [60]. Despite divergent morphologies, all autopodia are dorsoventrally flattened structures, as can be seen by juxtaposing the hands and feet of the evolutionarily distant and morphologically divergent lizard and mouse (Additional file 6). In instances where clefting occurs in mammals (human, Fig. 10e, f), the autopodium is bifurcated along the distal midline (ectrodactyly) and results in missing elements or a reconfigured architecture [34].

Despite having a relatively normal complement of phalanges and a pentadactyl number of digits, chameleons have an extremely modified carpus and tarsus (Fig. 10a-d). In addition, the proximal skeleton of the autopodium (the wrist and ankle, or mesopodia) is reduced in terms of the number of bone elements. Furthermore, those that remain are enlarged and form a ball-and-socket joint between the autopodium and forelimb (zeugopodium). Architecturally, clefting and syndactyly are necessary for grasping, but these two phenotypic novelties are not sufficient to explain the ball-and-socket mesopodial skeleton. Thus, wrist and ankle bone homologies remain contentious relative to outgroup lizards and other tetrapods [25, 61].

Previous investigations of the potential homology of derived mesopodial skeletal elements in chameleons relied on late gestation stage *Trioceros hoehnelii* and *Bradypodion pumilum* embryos [61–63]. Similarly, comparisons to outgroup lepidosaurs also relied on late gestation stage embryos and were also based solely on the number of visible ossification centers in the mesopodia. Consequently, distal carpals 2–4 were interpreted to have fused to make a large central element with the proximal forelimb elements comprising the radiale, ulnare and pisiform, while in the hindlimb only distal tarsal 4 and a ‘tarsale proximale’ were present [25, 62, 63].

To better understand autopodial evolution and structural diversity across chameleons, we generated a skeletal ontogenetic series of *Chamaeleo calyptratus* in order to: 1) identify the developmental sequence of appearance of autopodial skeletal elements, 2) homologize the derived and simplified elements of the chameleon autopodium, and 3) understand what developmental events occur in the formation of *C. calyptratus* autopodia.

**Fig. 11:**
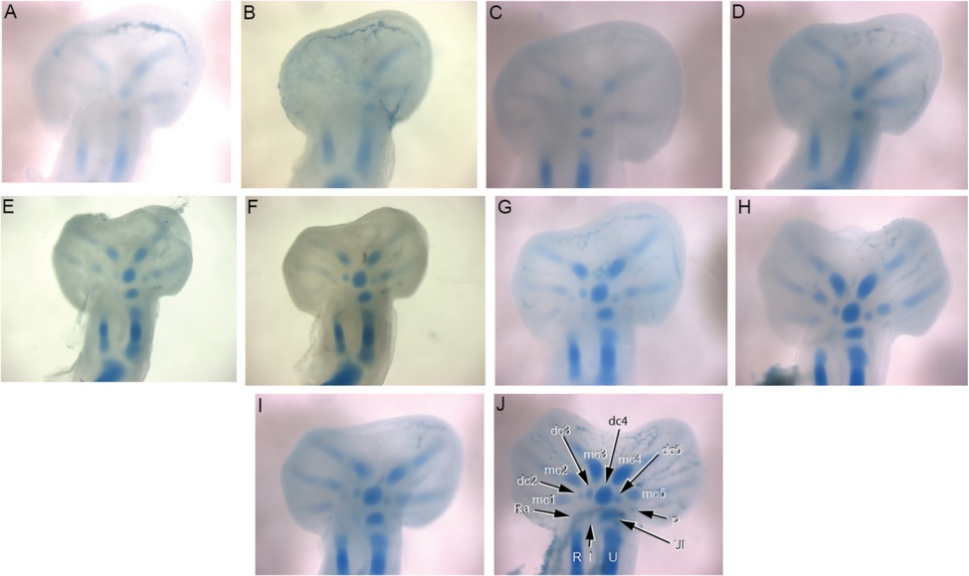
Chondrogenic differentiation in chameleon carpus. During distal flattening and expansion (**a**-**c**), the digital rays are already present and are lightly stained by Alcian Blue. The first elements of the mesopodium to form as cartilage are the Fi, dc4, and metacarpals 3–5. Distal carpal 3 and 5 form next (**d**, **e**) with later appearance of the intermedium, metacarpal 2 (**f**-**h**) and ultimately the radiale and pisiform (**i**-**j**). All embryos were at 107 dpo from clutchmates

**Fig. 12:**
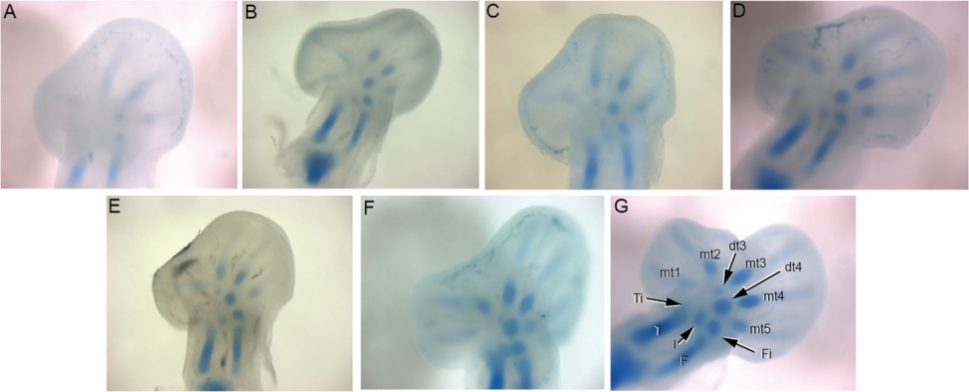
Chondrogenic differentiation in chameleon tarsus. During distal flattening expansion, the first elements to appear are the ulnare and distal tarsal 4 (**a**) followed by distal tarsal 3 and metatarsals 3–5 and the intermedium (**b-c**). Metatarsal 2 is followed along with an appearance of the tibiale (**e-g**). All embryos were at 107 dpo from clutchmates

**Fig. 13:**
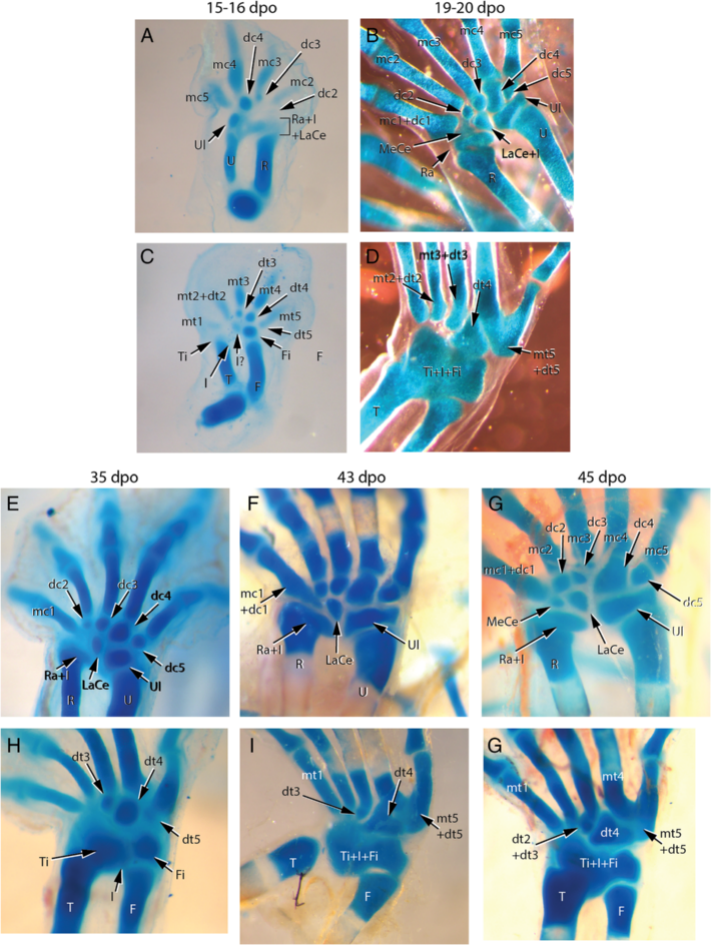
Outgroup lizard species autopodial chondrification. Desert grassland whiptail lizard (*Aspidoscelis uniparens*; Teiidae) and bearded dragon (*Pogona vitticeps*; Agamidae) chondrification patterns show post-axial dominance in elements appearance along the metapterygial axis. *A. uniparens* (**a, b**) hands show early formation of the ulnare, distal carpals 3 and 4, metacarpals 2–5 and a diffuse staining in the area of the radiale, lateral centrale and intermedium **a**. This empty domain later fills with a large medial and lateral centrale **b**. The foot develops from less elements and completes with a fused metatarsal 5 and distal tarsal 5 and a large distal tarsal 4 **c-d**. A large proximal tarsal element comprising the fibulare, intermedium and tibiale + lateral centrale develops in the ankle. In *P. vitticeps* **e-j**, a very similar sequence of cartilage elements in the hand (**e-g**) develop relative to *A. uniparens* with the added intermediate stage of seeing the medial carpale segment from the proximal end of distal carpal 1 (**f**) while the lateral centrale shifts distally adjacent to dc2 and dc3. The tarsus is more difficult to follow due to a small sample size and diffuse staining, but the proximal tarsal element is at least formed by the tibiale, intermedium and fibulare (**h-j**) with centralia not distinguishable

**Fig. 14:**
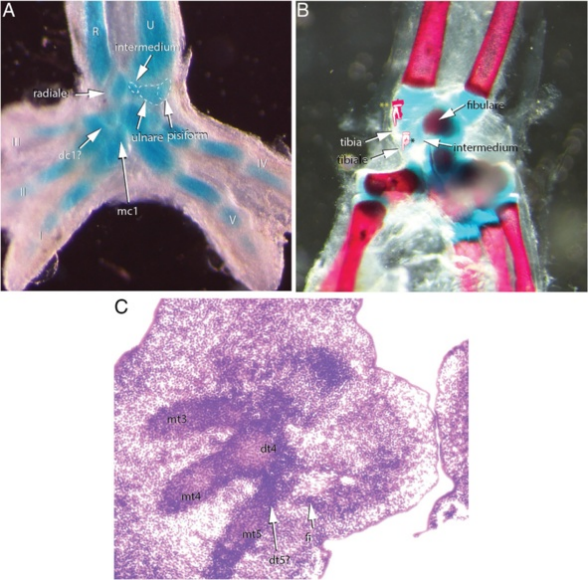
Contentious skeletal elements of the chameleon mesopodium. While broadly considered to be a sesamoid, we see the pisiform segmenting from the ventrolateral margin of the ulnare, underscoring its origin from the primary skeleton (**a**). In ‘true chameleons’ we see what appear to be ectopic and possibly *de novo* elements on the posterior tarsus. **b** The two distal elements are actually the tibiale and the intermedium which become shifted distally toward distal tarsals by the enlarged distal epiphysis of the tibia. **c** Despite the highly modified autopodium, chameleons retain distal tarsal 5

**Fig. 15:**
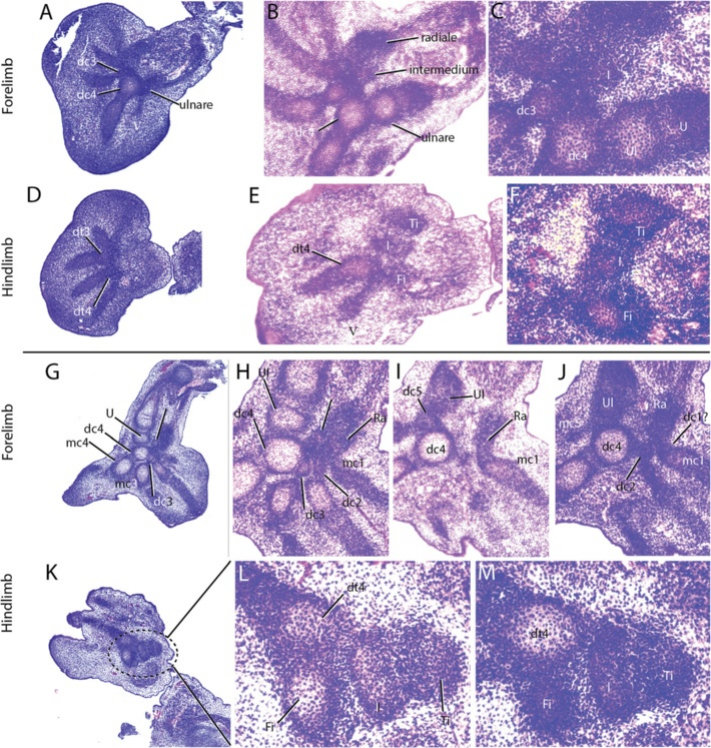
Histological examination of mesopodial mesenchymal and cartilaginous condensations. To complement the wholemount alcian cartilage stains, standard H&E paraffin sections were made to look for condensations which may not have shown due to a failure to reach the chondrification stage. In the carpals, we were able to find additional support for elements such as the intermedium, radiale and ulnare (**a**-**c**, **g**-**j**). In addition, we were able to identify a condensation which appears to be small and transient as distal carpal 1 (**j**) which does not chondrify and has a significant contribution of mesenchyme from the preaxial side of the limb. In the tarsals, we see a significant amount of shared mesenchymal cells between the distal tarsal 4, fibulare, intermedium and the tibiale (**d**-**f**), with the tibiale and intermedium always in closer association and smaller

**Fig. 16:**
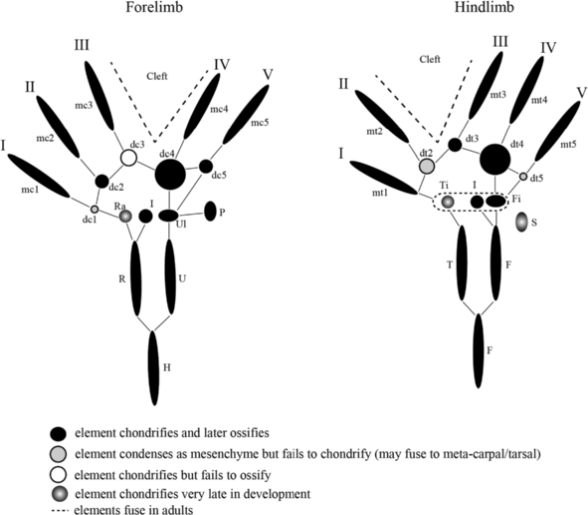
*Chamaeleo calyptratus* atuopodial skeletogenic condensations. Despite a very specialized and element reduced adult chameleon autopodial skeleton, most ancestral skeletal elements remained present through ontogeny with the exception of the centralia. Between the manus and pes, the preaxial elements were always significantly delayed (ra, ti) in appearance. The intermedium appeared to have a ‘dual’ origin, with its condensation occurring closer to the radius in the forelimb and the fibulare in the hindlimb. We found that elements condensed and completed skeletogenesis (metacarpals, at least half of the distal carpals/tarsals, pisiform). There was a high tendency for fusion amongst proximal elements of the foot in *C. calyptratus* despite delayed appearance of the tibiale. Distal carpal 1 and distal tarsal 2 were seen to condense as mesenchyme but later fuse to adjacent elements while we were unable to find any condensation for distal tarsal 1. Distal carpal 3 remains cartilaginous. The pisiform was found to be derived through segmentation of the ulnare and the fibularis brevis tendon to enclose a sesamoid

In our skeletal analysis of *Chamaeleo calyptratus*, we identified 2 rows of skeletogenesis during development of the carpals (Fig. 11a-j; Fig. 15a-c, g-j): 4 proximal carpal elements (pisiform, ulnare, intermedium, radiale) and 5 distal elements (distal carpals 1–5). Tarsal elements also developed from two rows (Fig. 12a-g; Fig. 14b, c; Fig. 15d-f, k-m): 3 proximal elements (fibulare, intermedium, tibiale) and 4 distal elements (distal tarsal 2–5; summarized in Fig. 16). Interestingly, the intermedium appears to condense in association with different mesopodial elements, from the distal medial radius in the hand and from the distal medial fibula in the foot (Fig. 15). Thus, *C. calyptratus* retains more mesopodial elements than previously acknowledged, with only the medial and lateral centrale of Lepidosauria failing to develop Additional file 2. Centralia are generally the last elements to form ontogenetically [64, 65] and are consistent with a heterochronic terminal truncation. A clear series of proximal condensations is not present in the outgroup taxa *Aspidoscelis uniparens* and *Pogona vitticeps*, as observed by the increased and more diffuse alcian blue staining present across this domain (Fig. 13).

During early embryogenesis, 9 carpal elements and 7 tarsal elements form in *Chamaeleo calyptratus* autopodia. The hands and feet at 114 dpo are dorsoventrally flattened, the plesiomorphic condition for tetrapods. During later stages of embryogenesis, fusion of proximal elements and a reorganization of the distal limb skeleton gives way to a distal cleft and zygodactyly. In the adult manus of *C. calyptratus*, the 4 proximal elements of the first row are retained, while only 3 distal carpal (dc) elements are present, dc2, (dc3 + dc4), dc5. In the pes, the fibulare, intermedium, and tibiale fuse, leading to a single ossification center comprising the proximal tarsal element (PTE), while distally, the distal tarsals (dt), dt2, dt3, dt4 remain as independent bony elements.

While observing the formation of the carpals, the ulnare was noted to undergo a segmentation event from its ventrolateral surface at 114 dpo (Fig. 14a). Subsequent analysis with later stage embryos provided evidence that this particular element later ossified as the pisiform bone. The pisiform is a large ossified element on the posterior margin of the manus in leaf mimic chameleons (*Brookesia*, *Rieppeleon*, *Rhampholeon*). The pisiform increases in size (both mediolaterally and in the development of a dorsolateral process) as one samples higher taxa across the chameleon phylogeny [66]. Thus, the origin of the pisiform as a sesamoid is not supported by our study (Additional file 3: Figure S3A, B; Additional file 5: Figure S5). Both the pisometacarpal ligament and the tendon of the flexor carpi ulnaris are visible with separate insertions, not enclosing the pisiform within a tendon as expected for a sesamoid in adult chameleons. This conclusion contrasts with a recent study which also recognized the pisiform’s origin from the ulnare, but declared it as a sesamoid [67]. The bony element of the posterior tarsus is widely considered a sesamoid but was not apparent until after hatching in our study of *C. calyptratus* (see Additional file 3: Figure S3C, D). This element clearly originates within a tendon (of the *fibularis brevis* inserting onto the proximal end of the hooked ‘5^th^ metatarsal’) and supports the effect of biomechanical stress in inducing the skeletogenic program of sesamoids [68]. Additional file 5: Figure S5A-C illustrates the segmentation of the pisiform from the ventrolateral wall of the ulnare while transverse histological sections show the pisiform segmenting from the ulnare with a shared outer condensed layer of mesenchymal cells, the prospective perichondrium (Additional file 5: Figure S5D-H).

#### Broad mesopodial skeletal diversity across Chamaeleonidae

To understand how skeletal fusions and heterochronic changes during skeletogenesis in the mesopodium of chameleons have been modified by natural selection, due to the need to adapt to an arboreal lifestyle, we sampled species representing 8 of the currently recognized 12 genera of extant chameleons [21, 66]. *Palleon* was examined from an embedded 3D microCT isosurface reconstruction in Glaw [69].

Despite an overall conserved body plan, chameleon body size ranges from the large tree adapted *Furcifer oustaleti* and *Calumma parsonii,* at approximately 68 cm total length, to the recently described *Brookesia micra*, whose maximum total length is less than 30 mm [22, 70], a striking 23 fold difference. Within the family Chamaeleonidae, 2 genera diverged early (*Brookesia* and *Palleon*) in Madagascar with all other genera forming the sister clade. The two Malagasy genera and two basal genera found within the sister clade are known as the Leaf Chameleons as they are leaf mimics, whose niche encompasses ground cover and low shrubs in Madagascar and Africa [66, 71]. Classically, morphological descriptions of chameleon hand and foot skeletons came from two derived and highly arboreal genera, *Trioceros* and *Chamaeleo* [25, 64, 72, 73], thus biasing our knowledge of morphological diversity of autopodia across chameleons. This has inadvertently led to an underappreciation of skeletal element number with respect to carpals and tarsals in chameleons generally.

Our comparative analysis illustrates that the most basal lineages of chameleons (and the two early diverged genera in Madagascar) maintain the least number of independent carpal and tarsal elements as adults (see Fig. 15). In the forelimb mesopodium, the lowest number of independent skeletal elements are found in the genera *Brookesia*, *Rieppeleon* and *Rhampholeon*. Members of these genera have a mesopodial formula of 3/1 (proximal row elements/distal row elements) representing a [radiale + (intermedium + Ulnare) + pisiform]/[fused dc 2–5]). All chameleons examined lack the middle row comprised of two centralia which are found in outgroup lepidosaurs. An isosurface microCT 3D rendering of *Palleon lolontany* grossly presents with the same autopodial morphology as *Brookesia* [69]. The foot mesopodial skeleton was greatly simplified in these three genera. The most reduced architecture is found in *Rieppeleon brevicaudatus*, having a single distal tarsal and two proximal elements of which one was a sesamoid within the fibularis brevis tendon, whereas the other was a fused tibiale + intermedium + fibulare. Surprisingly, in *Rieppeleon* the proximal face of all metatarsals fuse with adjacent metatarsals to form a single crown surrounding the distal tarsal element (Additional file 1: Figure S1E). *Brookesia* and *Palleon* were found to be the only taxa sampled lacking the *fibularis brevis* sesamoid ossification. This may be representative of basal chameleons [69] and/or locomotory mode.

From this study we can conclude that increased body length in chameleons >20 cm accompanies a corresponding increase in the number of carpal elements, from 3/1 (proximal row/distal row) in leaf mimic chameleons, to 3/2, 3/3, 4/3 and 4/4 in the phylogenetically derived ‘True Chameleons’. Hindlimb mesopodial skeletal elements also increase from 1/1 (0) [proximal 2 rows with sesamoid in parentheses) in leaf mimics, through 2/2 (1), 2/3 (1), 3/1 (1), with variation in *Chamaeleo* spanning 2/3 (1), 1/3 (1), and 2/1 (1). All but *Brookesia* and *Palleon* in this study retain a tarsal sesamoid. As in other lepidosaurs [74], the foot skeleton is always composed of fewer elements than the manus.

In the hindlimb mesopodium, we observed 1–2 elements which appeared to be ectopic or *de novo* structures in *Bradypodion*, *Chamaeleo*, *Furcifer* and *Trioceros*. Knowing that in *C. calyptratus* the proximal tarsal is initially composed of condensations for the tibiale, intermedium and fibulare (Fig. 12), we examined cartilage development in a 114 dpo *C. calyptratus* embryo (Fig. 14b) to follow the possible origin of these ectopic elements. At this stage, the distal epiphysis of the tibia has grown extensively in the distal direction and displaced the cartilages of the intermedium and the tibiale toward the metapodial skeleton. In adults (Additional file 1: Figure S1I), the tibiale, intermedium and fibulare fuse, forming a projection medially on the proximal tarsal element. The tibia is able to redirect the placement of the tibiale and intermedium from their proximal position. Using these results as a model, the distal mesopodial element in *Bradypodion* would be the tibiale and the proximal tarsal element would be comprised of a fused intermedium and fibulare.

Across the genus *Chamaeleo,* 0–2 ectopic elements are present distally while 2 are visible in both *Furcifer* and *Trioceros*. Using the distal epiphyseal growth of the tibia as a model for proximal tarsal elements as seen in *C. calyptratus*, it is evident that the distal elements in the former two genera are due to a more distal tibiale and a more proximal intermedium, with the proximal tarsal element composed of only the fibulare. Thus, distal displacement of the intermedium and tibiale is due to an accelerated growth heterochrony in the epiphysis as well as a loss of fusion for the three proximal tarsal elements which generally produce a single proximal tarsal. The proximal tarsal element in different chameleon taxa is also shown to have different compositions and are thus not homologous in our study.

#### Functional and phylogenetic constraints on mesopodial skeletal elements

Carpal and tarsal tunnels of the wrist and ankle, respectively, guide tendons, nerves and blood vessels from the zeugopod into the autopodial domain and are involved in flexion of the more distal skeletal elements. Chameleons, unlike other tetrapods, have an autopodial skeleton with an approximately 90 degree perpendicular fixed angle to the proximodistal axis of the appendicular skeleton (see Fig. 1a-c). In association with a highly modified ‘ball-and-socket’ architecture of mesopodial elements, tissues extending through the tunnels must not only be enclosed through retinacula but must also be inhibited from sliding laterally and out of the tunnel by digits 1 and 5 during wrist extension, the position of which is fixed in chameleons [75]. To compensate for the perpendicular orientation of the autopodium in chameleons, wrist elements of the hand (radiale, pisiform) have enlarged and grown ventromedially to laterally border the tunnel connective tissues. Furthermore, the large distal carpal element (of variable composition phylogenetically as discussed above; compare skeletal elements in posterior view in Additional file 1: Figure S1D-N) develops a ventral concavity to funnel the connective tissues to the palmar side toward the ventral aspect of the metacarpals (primarily in ‘leaf chameleons’).

**Fig. 17:**
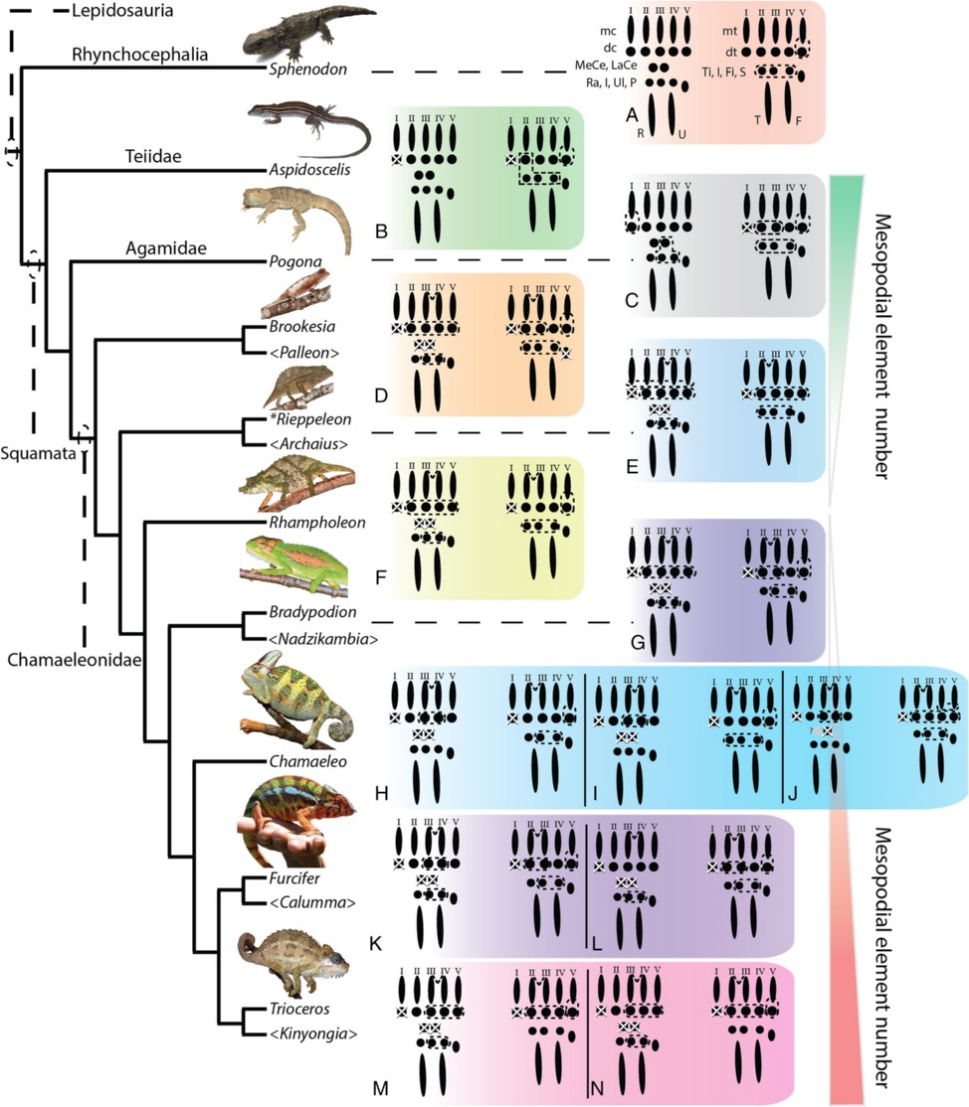
Evolutionary trends in chameleon autopodial morphology. Outgroup comparison was conducted with the extant Rhynchocephalian *Sphenodon punctatus* (**a**) and 2 outgroups within Squamata (Teiidae [*Aspidoscelis; * (**b**)], Agamidae [*Pogona; * (**c**)]). Fusion of proximal tarsal elements arose during amniote evolution by formation of the Astragalus (tibiale + intermedium) and the Calcaneum (fibulare) (see [87] for review). *Aspidoscelis* (**b**) and *Pogona* (**c**) both show an increase in fusion of mesopodial elements relative to *Sphenodon* (**a**). The small and early diverged chameleons (*Brookesia* (**d**), *Palleon*) show the distal clefting of the autopodia between the forelimb digits III and IV and hindlimb II and III characteristic of chameleons. These two genera also present the greatest modified proximal autopodial skeleton within chameleons. The larger *Rieppeleon* and *Rhampholeon* (with shorter tails; (**e**, **f**)) presents a sesamoid in the fibularis brevis tendon of the heel which is maintained in all higher chameleons. *Bradypodion* (**g**), *Chameleo* (**h**-**j**), *Furcifer* (**k**, **l**) and *Trioceros* (**m**, **n**) (‘true chameleons’) sampled in this paper showed an ascending phylogenetic trend toward increased body size and an associated increase in skeletal elements present in the mesopodium, leading to a wrist with increased flexion capability as well as being the first amniote group to form an independently ossified tibiale and intermedium which is only known to be present in amphibians. Thus, in early chameleon lineages there appears to be a skeletal bottleneck relative to outgroup squamates with a subsequent phylogenetic trend of ‘re-evolving’ mesopodial elements present in outgroup taxa. The red gradient shows a trend toward reduction of elements from outgroup taxa to chameleons while the red gradient shows a relative increase in mesopodial skeletal elements in advanced chameleon genera studied. Genera in “<*Genus*>” are currently recognized taxa but were not examined in this study. Letters within figure correspond to the species with the same letters in Additional file 1: Figure S1

In lepidosaurs, metatarsal 5 and distal tarsal 5 are fused [62] (Fig. 13c, d, h-j; Additional file 1: Figure S1; Fig. 12). Thus, a hooked ‘5^th^ metatarsal’ serves as a synapomorphy for this clade. In the chameleon foot, ‘mt5’ and mt1 bilaterally border the connective tissues through the tarsal tunnel to the plantar surface of the foot. Basal species exhibit a single proximal tarsal element and a large distal tarsal element. Phylogenetically derived genera (*Chamaeleo, Trioceros, Furcifer*) enclose the tarsal tunnel connective tissues by increasing the relative size of the fibularis brevis tendon while also making the tibiale and intermedium independent from the proximal tarsal element. This changes the composition of the proximal tarsal element across taxa in exchange for increased ventromedial support of the tarsal tunnel connective tissues (Fig. 17; Additional file 1: Figure S1D-N).

## Discussion

Chameleon autopodia have undergone extensive modification for optimized locomotion in a complex three-dimensional arboreal habitat. Here we have taken advantage of the slowly developing Veiled Chameleon (*Chamaeleo calyptratus*) [29] to characterize the morphogenesis of the squamate limb and study various aspects of morphogenesis of the hands and feet. This has aided our understanding of the diversification of autopodial morphologies across 8 of the 12 currently recognized chameleon genera and how the derived autopodial morphology of chameleons emerged within squamate reptiles. Chameleons exhibit a typical pentadactyl morphology, but also present with a midline autopodial cleft (ectrodactyly) and two opposable syndactylous bundles of highly mobile digits. In addition, the proximal skeleton of the autopodium in chameleons was modified *via* a reduction in the number of bone elements.

Due to the presence of 5 digits in chameleons, the ancestral condition for tetrapods, we expected a conserved pattern of expression for both *Fgf8* and *Shh* in the developing autopodia of chameleons. Indeed, *Fgf8* was observed in the Apical Ectodermal Ridge (AER) while *Shh* was present in the Zone of Polarizing Activity (ZPA) from the onset of limb bud outgrowth through termination of distal limb outgrowth and morphogenesis. All previous studies of autopodial ectrodactyly and clefting have concluded this phenotype as having occurred due to a loss of integrity of the AER. In contrast, we show that in chameleons, the AER remains a robust ectodermal thickening and expresses *Fgf8* during proximal mesenchymal clefting.

Interdigital cell death, also an ancestral condition for tetrapods, was mainly observed in chameleon autopodia between the digit pairs undergoing clefting while adjacent digits retained interdigital mesenchyme (syndactyly). Interestingly, inhibition of Bmp signaling prior to the initiation of interdigital cell death prevented cleft formation. However, upon the commencement of cleft formation, blocking Bmp signaling has no effect on interdigital cell death. Cell death between syndactylous digits was present in chameleons, but at significantly lower levels relative to the clefting mesenchyme. The syndactyly observed in chameleons may differ to that observed in duck feet and bat wings due to the absence of *Grem1*, a Bmp antagonist, in interdigitial tissue.

While previous studies credited interdigital cell death along the autopodial midline for the modified chameleon limb morphology, we highlight the previously unstudied and underappreciated skeletal diversity present in the mesopodial architecture across Chamaeleonidae. While retaining the ancestral pentadactyl morphology together with the typical (for squamates) phalangeal formulae, the chameleon wrists have been highly modified to have a reduced number of skeletal elements with subsequent enlargement of those elements that are maintained. Proximal carpals and tarsals of digits 1 and 5, respectively, of the hands and feet also make contact with each other. We have shown that during embryogenesis in *C. calyptratus*, the carpals comprise 9 skeletal elements and the tarsals 7, each of which originate as mesenchymal condensations, chondrify, ossify or fuse with adjacent elements at various stages of skeletogenesis.

We have used this model from *C. calyptratus* to homologize carpal and tarsal structures in 8 of the currently recognized 12 chameleon genera and found that chameleon research has significantly underestimated mesopodial skeletal diversity. The earliest diverged genera *Palleon* + *Brookesia*, and *Rieppeleon* are not only the smallest members of the family, but also show the greatest degree of skeletal number reduction in the wrist and ankle. More phylogenetically derived chameleon genera from this study *Chamaeleo*, *Trioceros*, *Furcifer* (‘true chameleons’) show the greatest number of independent skeletal elements present in the wrist and ankle within the family. This pattern also most closely resembles the skeletal complement found in outgroup lepidosaurs.

**Fig. 18:**
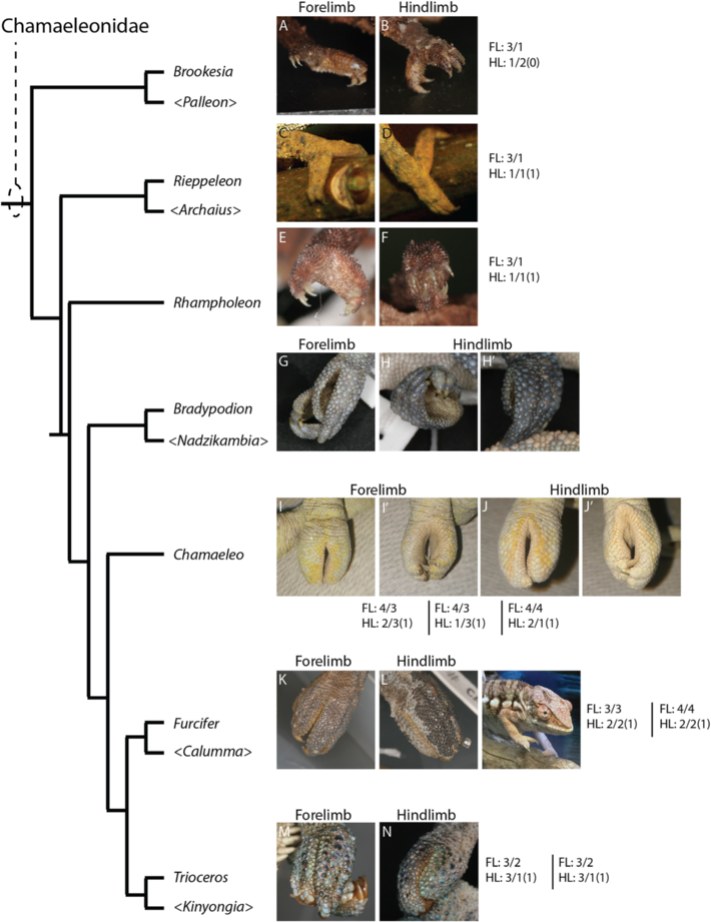
Phylogenetic reversal to a ‘typical’ lizard conformation of the autopodia. Despite retaining the cleft, an increase in mesopodial elements leads to increase wrist flexion in higher chameleons. Concomitant with the increase in the number of carpal and tarsal elements, we see a bifurcation where the syndactylous bundles are almost 180° separate in the early diverged lineages (‘leaf chameleons’; *Brookesia* (**a**, **b**), *Rieppeleon* (**c**, **d**), and *Rhampholeon* (**e**, **f**)) with the angle decreasing in *Bradypodion* (**g**, **h**, **h’**). ‘True chameleon’ syndactylous bundles shift toward the distal midline as is present in the basal squamate plan (with the cleft remaining as the constraint). While *Chamaeleo* (**i**-**j**) show an anteromedial transition of digit bundles, *Furcifer* (**k**, **l**) and *Trioceros* (**m**, **n**), the taxa with greater number of carpal and tarsal elements in this study, exemplify this the best through a greatly reduced angle between syndactylous bundles as digits move closer together along the distal midline. FL: refers to Forelimb with HL: referring to hindlimb. The numbers associated with each lineage on the phylogeny correlate to the same order of illustrations from Fig. 15 and represent formulae for number of elements present in the mesopodium in association with a representative image for autopodial morphology of a preserved specimen. FL: X/X refers to the number of elements in the two mesopodial rows while FL: X/X(X) refers to the two mesopodial rows with the number in parentheses representing the presence or absence of the sesamoid of the fibularis brevis. No centralia are present in chameleons, which would have made the standard 3 rows of mesopodial elements. Genera in “<>” are currently recognized taxa, but were specimens were not obtained in this study

A phylogenetic trend of gigantism appears to potentially correlate with the ‘re-evolution’ of mesopodial skeletal elements, as this is associated with an increased number of cells from which to build mesenchymal condensations in the mesopodia. An increase in mesopodial elements in ‘true chameleons’ also changed the resting posture of the hand and foot of chameleons, resembling a more typical lizard position where the two syndactylous bundles are oriented more anteriorly along the midline but are constrained due to the retainment of a cleft (Fig. 18). Chameleons therefore provide an important example of how heterochrony can modify developmental patterns while being constrained by functional requirements and possibly body size. Thus, extrinsic and intrinsic factors are selected by nature to produce highly adaptive morphologies.

Loss of carpal and tarsal elements increases the angle across the cleft zone, with the greatest angle being present in the most basal and smaller genera. More derived chameleon genera have an independent intermedium in the ankle, a structure which has not been described in any extant terrestrial amniote since the origin of the astragalus and calcaneum from the amphibian skeletal complement. In the wrist, segmentation takes place along the ventrolateral wall of the ulnare and gives rise to the pisiform bone. In contrast to the sesamoid bone of the fibularis brevis tendon of the hindlimb, the pisiform appears to originate with the primary endochondral skeleton and not within a tendon.

The family Chamaeleonidae is thought to have originated through the heterochronic process of paedomorphosis [76], with adults resembling late gestation embryos of more distant taxa [70]. Malagasy species belonging to *Palleon* and *Brookesia* range in total length from 2–10 cm [70]. Here we have identified that early diverged and basal genera (‘leaf mimic’ chameleons; *Palleon, Brookesia*, *Rieppeleon* and *Rhampholeon*) exhibit a dramatic reduction of independent carpal and tarsal elements in adults despite maintaining a normal pentadactyl number of digits and phalanges. This is in contrast to salamanders of equivalent size which exhibit digit loss and mesopodial element reduction [77–79]. While we only sampled 8 of the 12 extant genera (see Additional file 7), an ascending phylogenetic trend of increased body size appears to correlate with an increase (‘re-evolution’) in mesopodial element number to a complement close to that of outgroup lepidosaurs. More components in the wrist and ankle may have facilitated increased wrist flexion and provided a biomechanical advantage which has allowed more derived chameleons to leave the ground cover and low bushes for the trees.

Clefting of the autopodium *via* interdigital cell death is the ancestral condition in the tetrapod limb. In chameleons, interdigital tissue is however retained between fingers neighboring the midline cleft. This represents the derived condition. In phylogenetically more derived chameleon lineages with ‘re-evolved’ mesopodial skeletal elements, the ancestral tetrapod condition where all digits are present along the distal midline of the limb (Fig. 18) is almost achieved. While clefting separates the syndactylous bundles of digits, it is clear from the transiently bent chondrified digital rays of the alcian prepared skeletons (Fig. 11a-d; Fig. 12a-d) that there is either a musculoskeletal component driving the split, pulling the digit bundles toward the anteroposterior poles, or there may be a proliferative phase in the mesenchyme prior to cleft formation by apoptosis. Mesopodial elements have not received as much attention in the development literature when compared to studies of digit loss and phalangeal reduction.

Despite the three major subdomains of the limb (Stylopod, Zeugopod, Autopod) being highly conserved across tetrapods, species-specific differences in the morphometric dimensions of long bone elements of the zeugopodia may elicit a direct effect on carpal and tarsal elements. Broader elements (such as the radius and tibia) would provide a wider (anteroposterior) dimension of initial mesopodial mesenchymal pioneer cells for the more distal limb structures. We hypothesize that altered proximal limb width, especially in the zeugopodial domain, may affect chameleon evolution as a narrowing of this domain would ultimately provide a reduction in the distal mesenchymal cells available for building the mesopodial and acropodial skeleton. This model may also have been important for the evolution of digit and carpal reduced taxa such as *Hemiergis* [18] as they, like chameleons, have qualitatively thinner, elongated limbs with a smaller distal radius in zeugopodial skeletal elements. Larger taxa such as mammals, which lack a bottleneck in cell number for the mesopodial element and acropodial modification, employ genetic mechanisms for modifying adult phenotypes [13, 14, 80]. In contrast, small ectotherms appear to exploit the cell number constraint model and typically exhibit element size and element number variation. Within this framework, a smaller mesopodial mesenchymal domain would be expected to give rise to a reduced number of carpal/tarsal elements as well as fewer cells able to contribute to Digit 1. Notably, while two different studies [18, 80] have demonstrated a temporal reduction in *Shh* expression in association with digit reduction, the tetradactyl pattern in these studies was different. In the mouse, the digits were identified as having the character of digits I, II, IV, V, whereas the digit reduced lizard retained digits II, III, IV, V together with a reduction in mesopodial elements. Digit I loss in *Hemiergis* is consistent with our hypothesis that this phenotype is associated with a smaller initial pool of mesopodial mesenchymal cells. Fusion and reduction are more prevalent in organisms of smaller size, as was seen in this study, and this may simply be due to fewer mesenchymal cells being available for individual condensations to form or reach a sufficient density required for establishment of the chondrogenic pathway [49, 81, 82].

### Mesopodial elements do not ‘re-evolve’ *de novo*

Dollo’s Law on the irreversibility of complex traits has recently been challenged in taxa where autopodial elements appear to have been re-evolved [83, 84]. Phylogenetic trends in chameleon autopodial elements are not due to *de novo* ‘re-evolution’ of skeletal elements, but rather the elaboration of skeletal condensations already present. In members of the genera *Chamaeleo*, *Furcifer* and *Trioceros* sampled, mesopodial elements appear to be present but are underdeveloped or fused to adjacent condensations or skeletal elements, character states which can be easily missed when a detailed developmental series is not available for study. Mammalian species appear to reduce autopodial elements through genetically controlled cell death [12–14, 41, 80]. While a reduced period of SHH expression in the ZPA may also decrease the population size of mesenchymal cells available for autopodial development [18], narrow limbs such as those in *Hemiergis* lizards may have played an equally important role in reducing the cellular pool leading to element reduction [85]. Small mesenchymal population pools may also be a possible constraint on other members of the family Scincidae such as *Brachymeles* [86] and *Larutia* [87]. More laboratory and field studies are needed to test this.

Differential fusion between mesopodial condensations in chameleons blurs their skeletal homologies with outgroup lepidosaurs, especially if the topographic position of an element of interest or the number of ossification centers present are the only criteria considered. Herein we show that interspecific variability exists in not only the number of elements present (Figs. 16, 17 and 18) but also that different combinations of mesopodial elements fuse to give rise to the adult carpal and tarsal complement along with distal migration of other elements (intermedium and tibiale moving distally; see next section). Caution must be exercised when interpreting skeletal morphology using only dry skeletal specimens and microCT imaging, as neither allows for observation of cartilaginous tissue [88]. We have observed that fused condensations do not necessarily have multiple ossification centers, as individual condensations remain modular (despite fusing) and arrest their skeletogenic program at the mesenchymal, chondrogenic or ossification stage.

#### Homology and evolution of the Astragalus and tarsal bones

A proximal reduction in the number of carpal and tarsal elements in adult chameleons, relative to embryos and outgroup taxa, together with distal clefting, have allowed for evolution of the novel chameleon hand and foot which are used for complex grasping in an arboreal three dimensional environment. The progressive addition (‘re-evolution’) of skeletal elements in higher taxa may have facilitated increased flexion and a greater ability to grasp, which correlates with chameleons invading the arboreal niche [89] where only mammals with autopodial modifications such as marsupials have evolved to inhabit [90].

Chameleons are thought to have invaded Madagascar through two independent events [66]. The initial invasion by the ancestor of *Brookesia* and *Palleon* evolved *in situ,* and was followed by a secondary invasion of the ancestor of *Calumma* and *Furcifer* by dispersal from Africa. *Calumma* + *Furcifer* (Madagascar) are the sister lineage to *Trioceros* + *Kinyongia* (Africa), which collectively comprise the four most phylogenetically derived extant chameleon genera [66]. From our studies of the mesopodial architecture of these African and Malagasy clades, we can infer that the dispersed ancestor of *Calumma* + *Furcifer* to Madagascar was already mesopodially ‘pre-adapted’ to be an advanced climber due the larger complement of skeletal elements in the wrist and ankle. *Brookesia* and *Palleon* were already in Madagascar as forest floor dwellers. Given the advanced nature of the wrists in *Calumma*, *Furcifer*, *Trioceros* and *Kinyongia*, such increased mesopodial flexion has allowed them to comprise ~57 % of the 200 extant chameleon species [91].

Innovations associated with the evolution of terrestrial cursorial ability in amniotes involved modifications to the ankle. Evolution of the astragalus has elicited much discussion with recent models attempting to ascribe it an accurate homology with respect to tarsal skeletal elements found in anamniotes, generally involving 1–4 ossification centers (reviewed in [92]). Our work reveals a high degree of variability in the composition of the chameleon and squamate astragalus prior to the generally accepted co-ossification step following mesenchymal condensation. Chameleons of the genera *Chamaeleo*, *Furcifer* and *Trioceros* as shown here, are currently the only known amniotes to have successfully ‘re-evolved’ an independent intermedium and tibiale. A free intermedium and tibiale was lost in amniotes during the tetrapod progression onto land which selected for innovation of the astragalus and calcaneum [11]. Reptiles are considered to not have a tibiale (see references in [53, 93]). This ‘re-evolution’ of an ancestral series of tarsal elements may be due to a loss in selective pressure toward the typical terrestrial mode of tetrapod locomotion, in combination with a shift toward arboreality. Within this framework, different amniote lineages should be expected to develop an astragalus through the fusion of tarsal elements at different points through ontogeny. Although each element is modular, the distinct stage of skeletogenesis prior to and after fusion may vary across Squamata (Figs. 13 and 17).

In summary, examining the development of innovations and novelties in natural groups such as chameleons can further our understanding of how body plans are molded by natural selection as optimal phenotypes through adaptive evolution.

## Conclusion

Studying organisms in the lab with unique morphologies complements biomedical studies of normal development and congenital malformations while also providing insight into the limits (constraints) and novel deployment of molecular genetic pathways during embryonic development. Veiled chameleons (*Chamaeleo calyptratus*) present a highly derived body plan within tetrapods due to their specialization for living in an arboreal environment while also presenting a very slow rate of embryonic growth [27–29]. Developmental analysis of the specialized and bifurcate autopodia of *C. calyptratus* using histological, *in situ* hybridization and *in vivo* techniques show that the specialized mesopodial skeleton (wrist and ankle) forms despite the expression of highly conserved patterning genes during limb outgrowth. Our detailed skeletal developmental sequence present an unexpected doubling in the number of recognized wrist and ankle elements which has allowed us to homologize mesopodial elements across 8 of the 12 recognized chameleon genera. Our data also present a correlation between body size and skeletal diversity of the wrist and ankle during chameleon evolution with phylogenetically higher taxa exhibiting an increase in mesopodial skeletal element number, larger body size, and increased climbing ability. Exploring development in a slow developing taxon has also allowed us to observe the fine progressive development and segmentation of the pisiform from the ulnare in the hand and differential fusion of proximal tarsal elements in the ankle.

## Additional files

Additional file 1:
**Figure S1.** Comparative morphology of autopodia in this study. Dorsoventrally flattened autopodia in outgroup taxa (*Sphenodon punctatus, Aspidoscelis uniparens, Pogona vitticeps*; A, B, C) were drawn in dorsal view only (with *Sphenodon* illustrations adapted from [72] and [94]) for both forelimb and hindlimb autopodia. D-N are chameleon illustrations from taxa cleared and stained in this study to look for ossified and chondrified elements comprising the autopodial architecture and are illustrated in both Anterior/Dorsal and Posterior/Ventral views. A) *Sphenodon punctatus* (Rhynchocephalia), B) *Aspidoscelis uniparens* (Teiidae), C) *Pogona vitticeps* (Agamidae), D) *Brookesia stumpffi*, E) *Rieppeleon brevicaudatus*, F) *Rhampholeon boulengeri*, G) *Bradypodion pumilum*, H) *Chamaeleo dilepis*, I) *Chamaeleo calyptratus*, J) *Chamaeleo chameleon*, K) *Furcifer lateralis*, L) *Furcifer pardalis*, M) *Trioceros ellioti*, N) *Trioceros hohnelii*. (PDF 2863 kb) Additional file 2:
**Figure S2.** Forelimb condensations of *A. uniparens* in H&E stained sections. A large contribution of preaxial mesenchymal cells contributing to digit 1/distal carpal 1 are visible in these sections as well as the development of an independent Lateral Centrale and Intermedium at an older stage. The Medial Centrale will later appear as a medial segmentation from the distal carpal 1. (TIFF 4374 kb) Additional file 3:
**Figure S3.** Pisiform *versus* Sesamoid identity in the chameleon autopodium. The top row (A, B) shows the forelimb with the pisiform bone which we have identified in this study as segmenting from the ulnare. This bony element is not present within a tendon and instead has a proximal insertion of the tendon of the flexor carpi ulnaris with a more distal pisometacarpal ligament. The lower row (C, D) shows the hindlimb with a large sesamoid present *within* the tendon of the fibularis brevis which inserts onto the proximal margin of the ‘5^th^ metatarsal.’ The species used for this image was *Trioceros ellioti* (CAS 201725). (TIFF 7809 kb) Additional file 4:
**Figure S4.** Whole embryo culture of *C. calyptratus*. Details for this protocol are provided in the article text in the Methods section and correlate with labeled figures. (TIFF 7284 kb) Additional file 5:
**Figure S5.** Pisiform formation in the manus of *C. calyptratus*. Pisiform development from the ulnare is shown in posteroventral (A), lateral (B), and dorsal view (C) in whole alizarin/alcian skeletal preparations. Transverse H&E stained paraffin sections show, from distal to proximal mesopodium, the shared mesenchymal cells between the ulnare and the pisiform, supporting the ventrolateral segmentation of the pisiform from the ulnare (D-H). (TIFF 5828 kb) Additional file 6:
**Figure S6.** Autopodia are dorsoventrally flattened Divergent tetrapods such as the common lab mouse (Mammalia) and the desert grassland whiptail lizard (Reptilia) have divergent hands (A, C) and feet (B, D). Despite architectural differences, they remain dorsoventrally flattened. (TIFF 1825 kb) Additional file 7:
**Chameleon specimen measurements.** (DOCX 74 kb)

## Abbreviations

*Grem*: Gremlin
*Bmp*: Bone Morphogenetic Protein
*Shh*: Sonic Hedgehog
*Fgf*: Fibroblast Growth Factor
ICD: Interdigital Cell Death
AER: Apical Ectodermal Ridge
ZPA: Zone of Polarizing Activity
dpo: days post oviposition
PTE: Proximal Tarsal Element
SVL: Snout-Vent Length
TL: Tail Length
FL: Forelimb
HL: Hindlimb
Roman Numerals I-V: Represent digit 1- digit 5
Ra: Radiale
R: Radius
U: Ulna
Ul: Ulnare
P: Pisiform
I: Intermedium
T: Tibia
Ti: Tibiale
F: Fibula
Fi: Fibulare
dc: Distal carpal
dt: Distal tarsal
mc: Metacarpal
mt: Metatarsal
LaCe: Lateral centrale
MeCe: Medial centrale
