# Supplementary material for: Hand/foot splitting and the ‘re-evolution’ of mesopodial skeletal elements during the evolution and radiation of chameleons
Source: BMC Evol Biol. 2015 Sep 18;15:184. doi: 10.1186/s12862-015-0464-4 (PMC4574539; doi:10.1186/s12862-015-0464-4)

A) *Sphenodon punctatus*

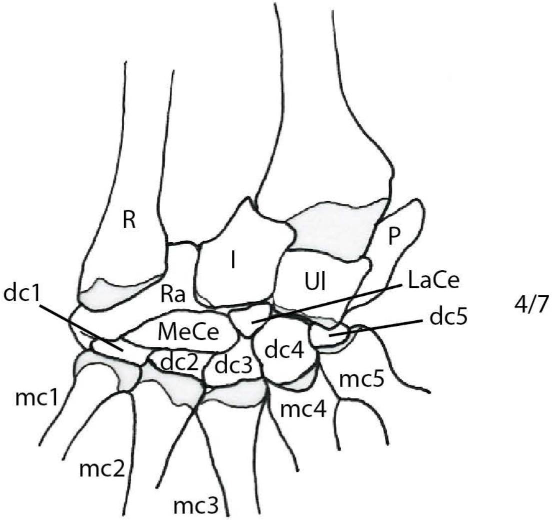

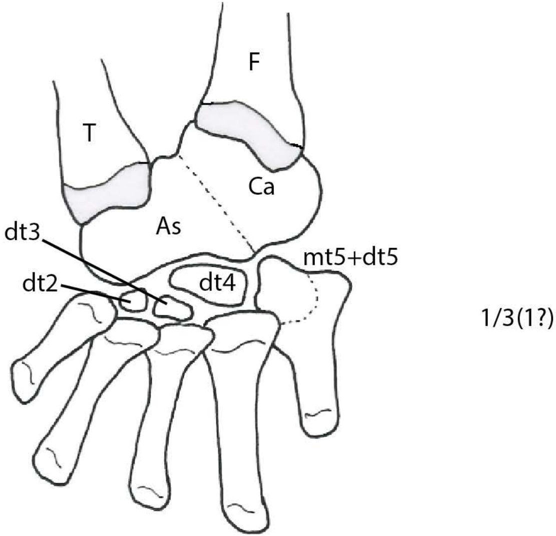

B) *Aspidoscelis uniparens* (hatchling)

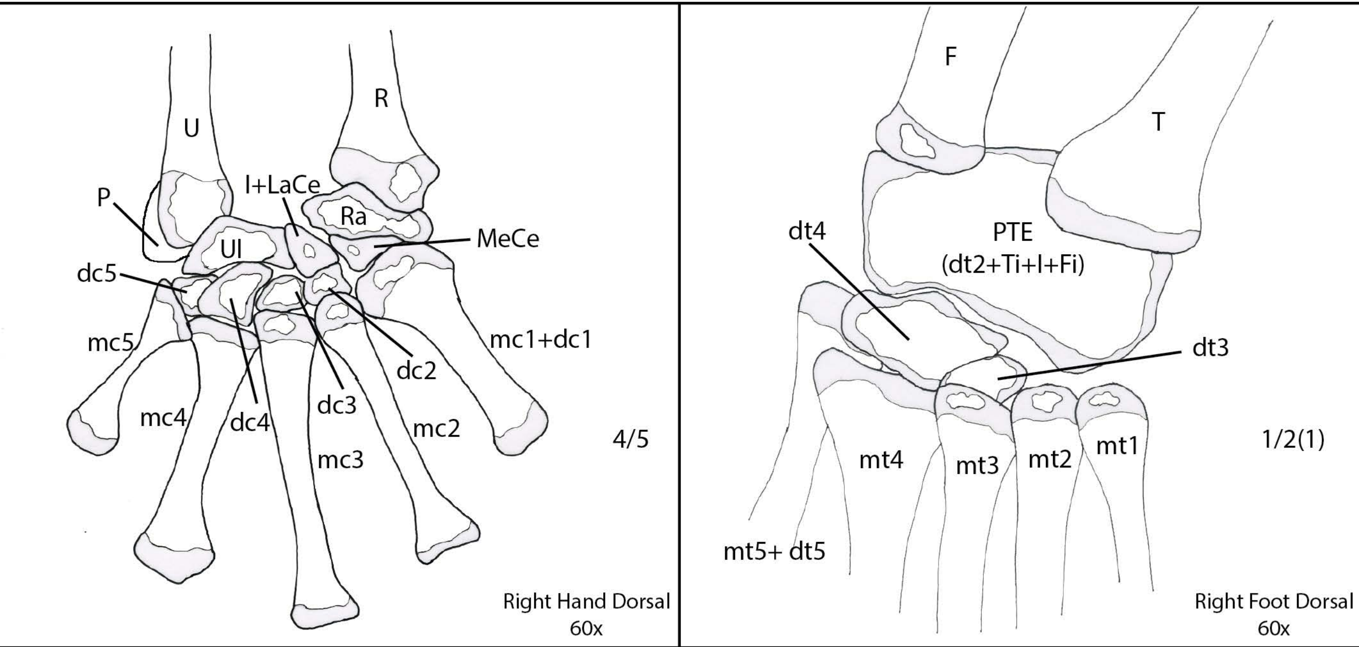

C) *Pogona vitticeps* (juvenile)

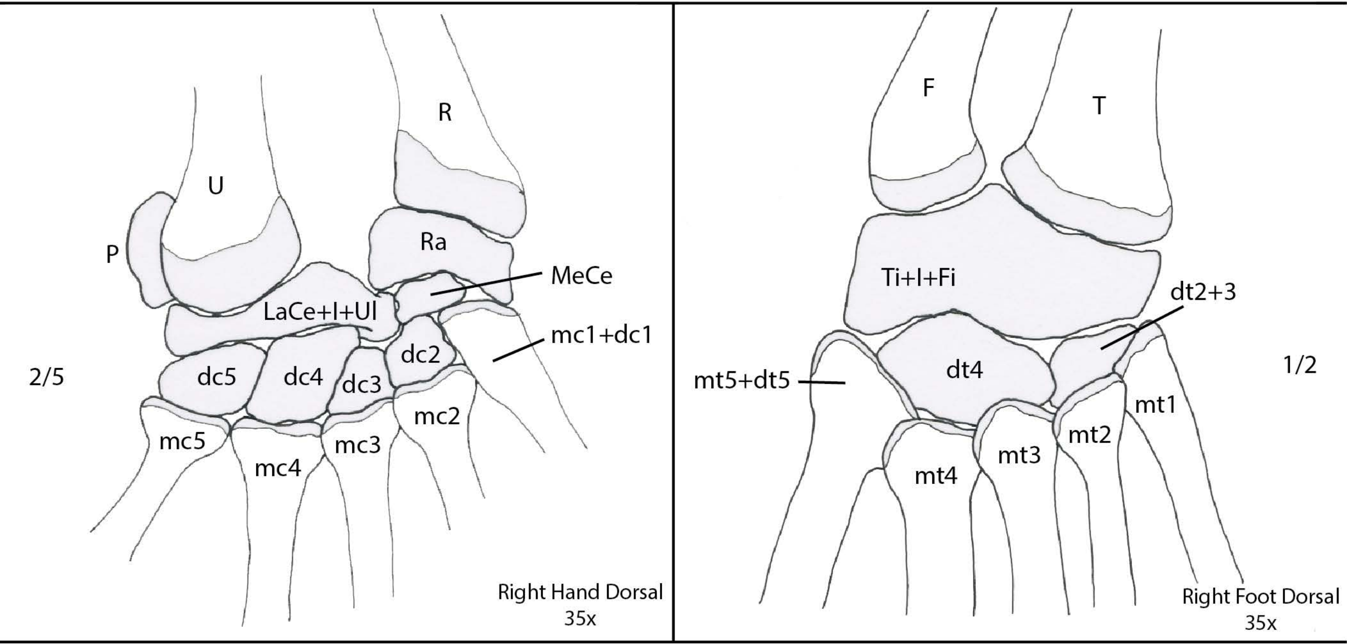

D) *Brookesia stumpffi* CAS 156861

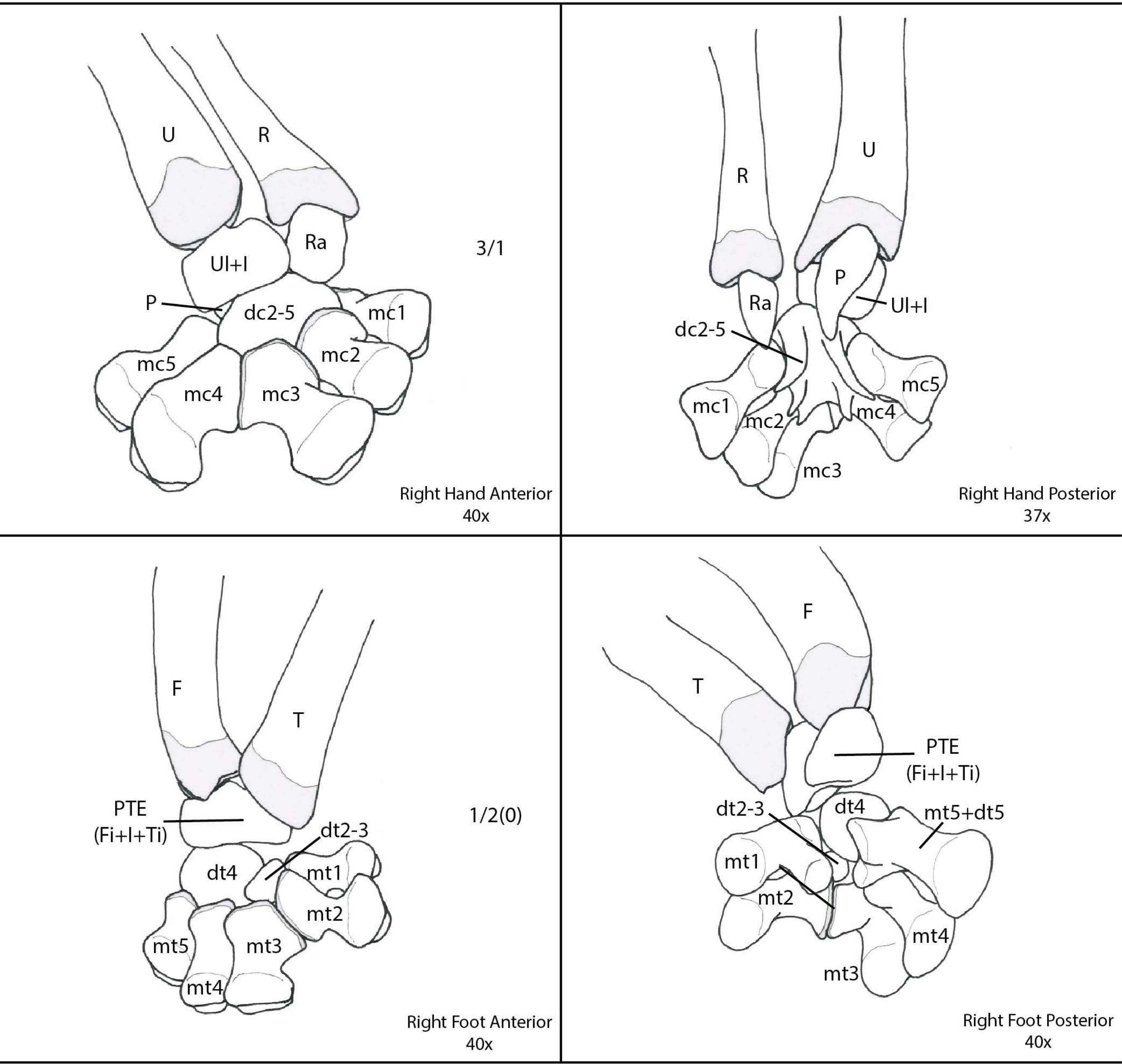

E) *Rieppeleon brevicaudatus*

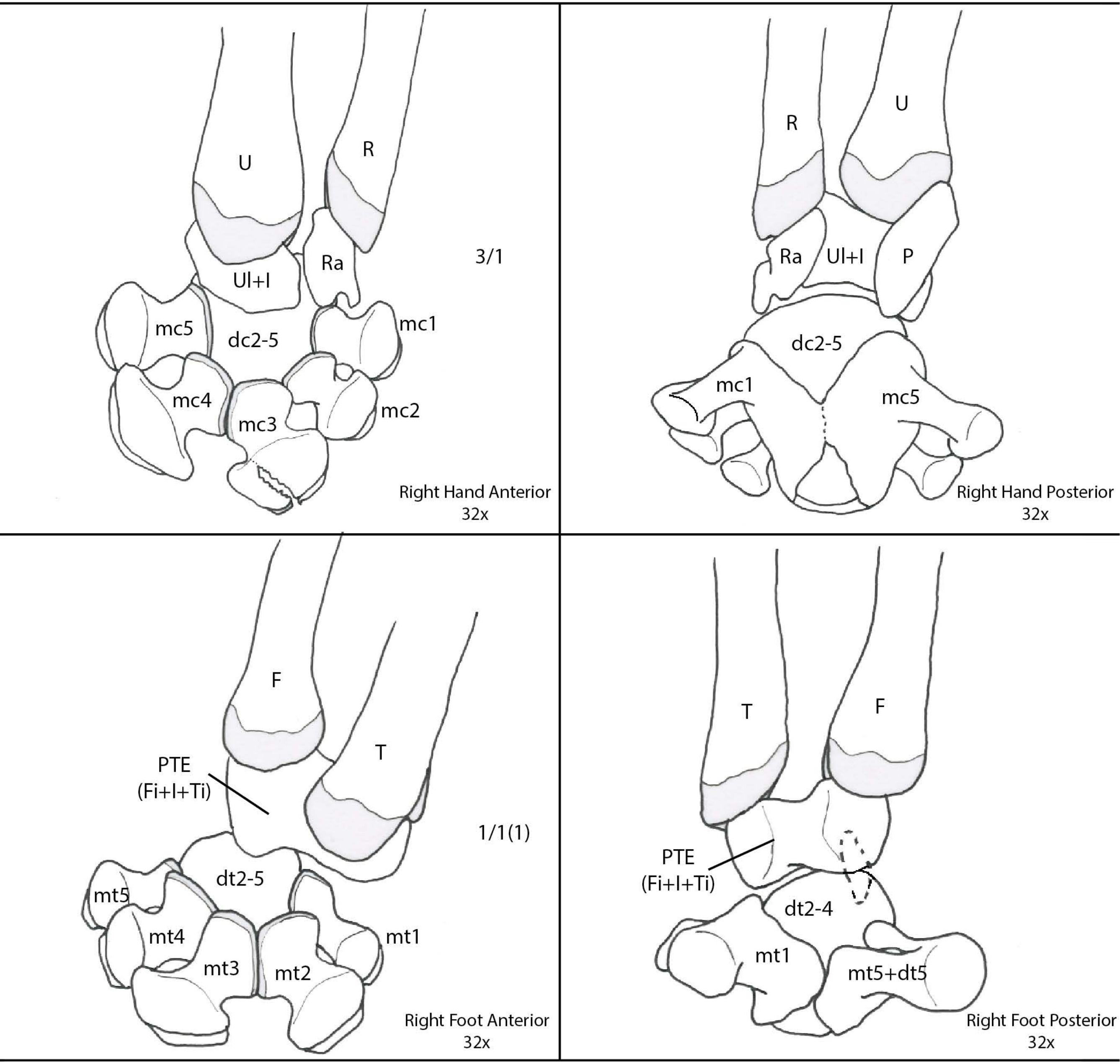

F) *Rhampholeon boulengeri* CAS 176860

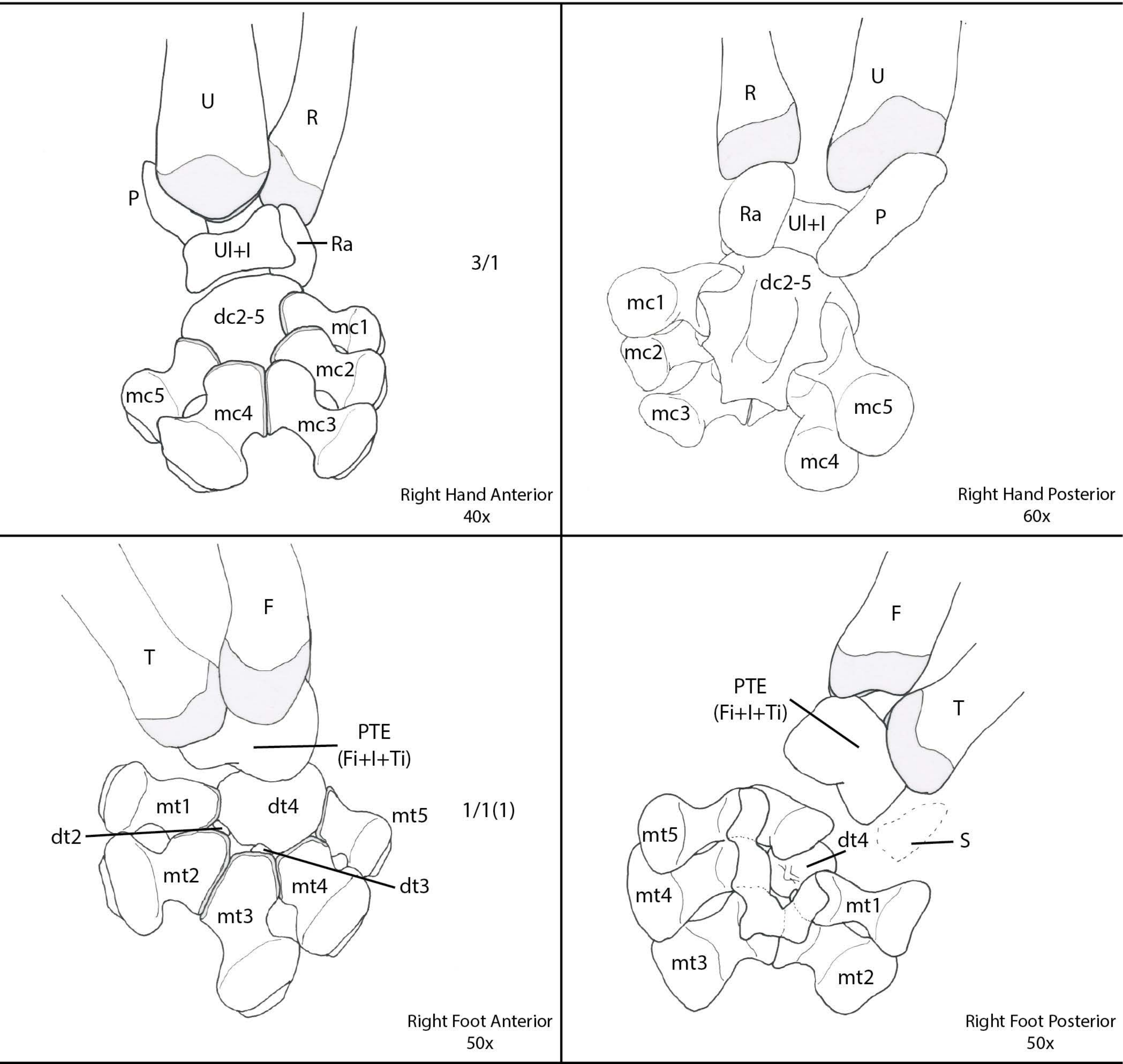

G) *Bradypodion pumilum* CAS 167754

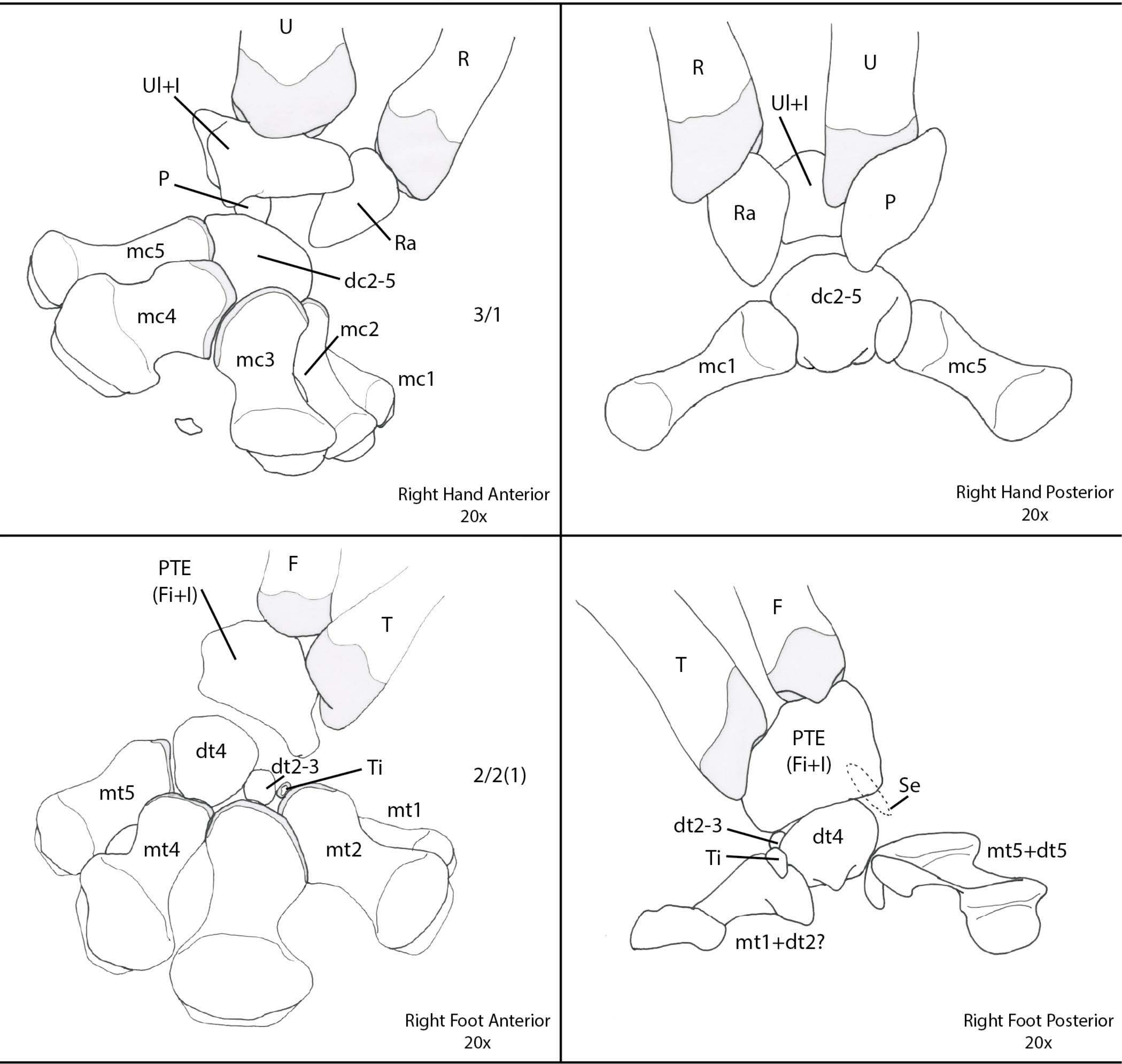

H) *Chamaeleo dilepis* CAS 54687

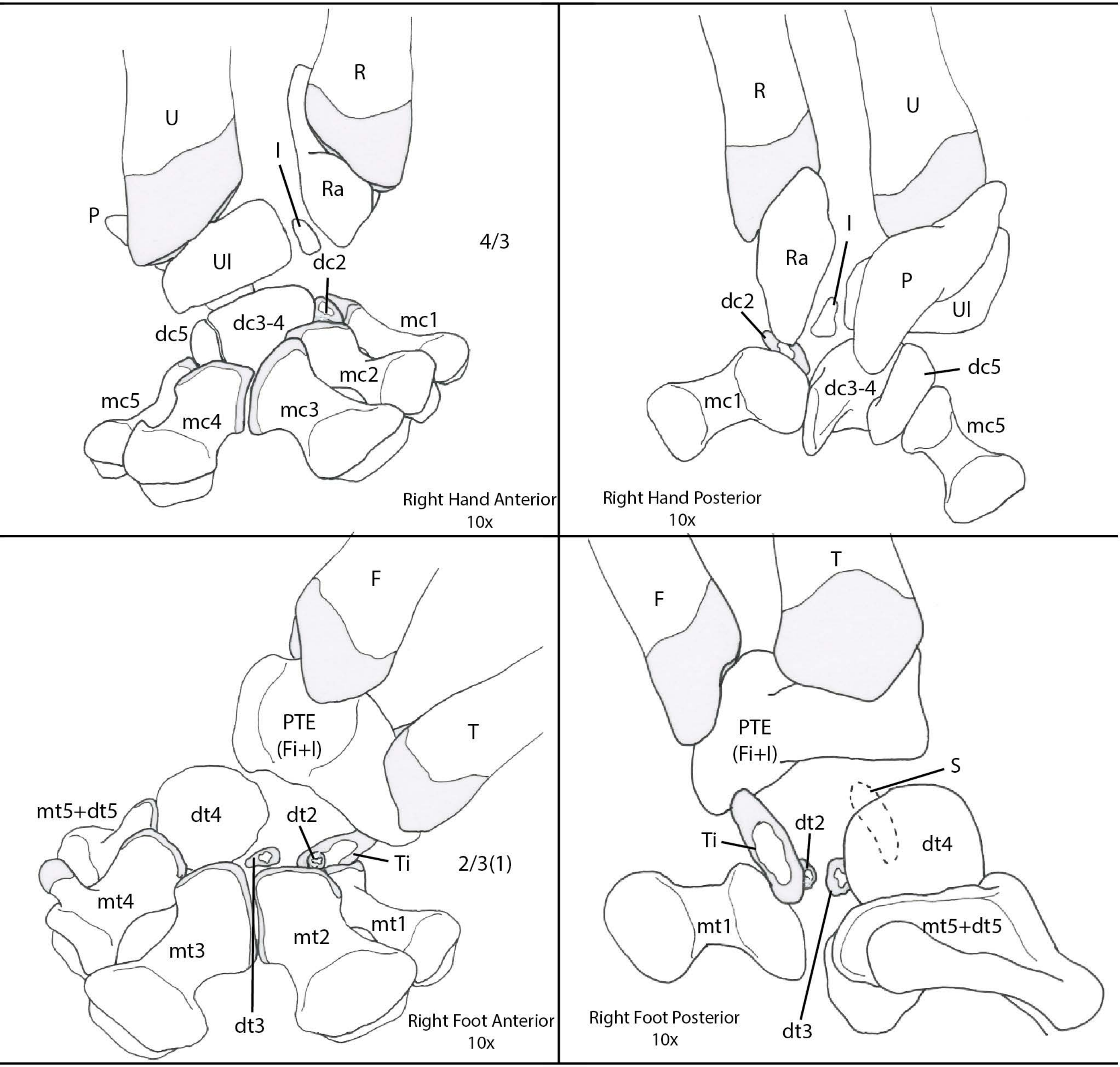

I) *Chamaeleo calyptratus* 7dph male #2

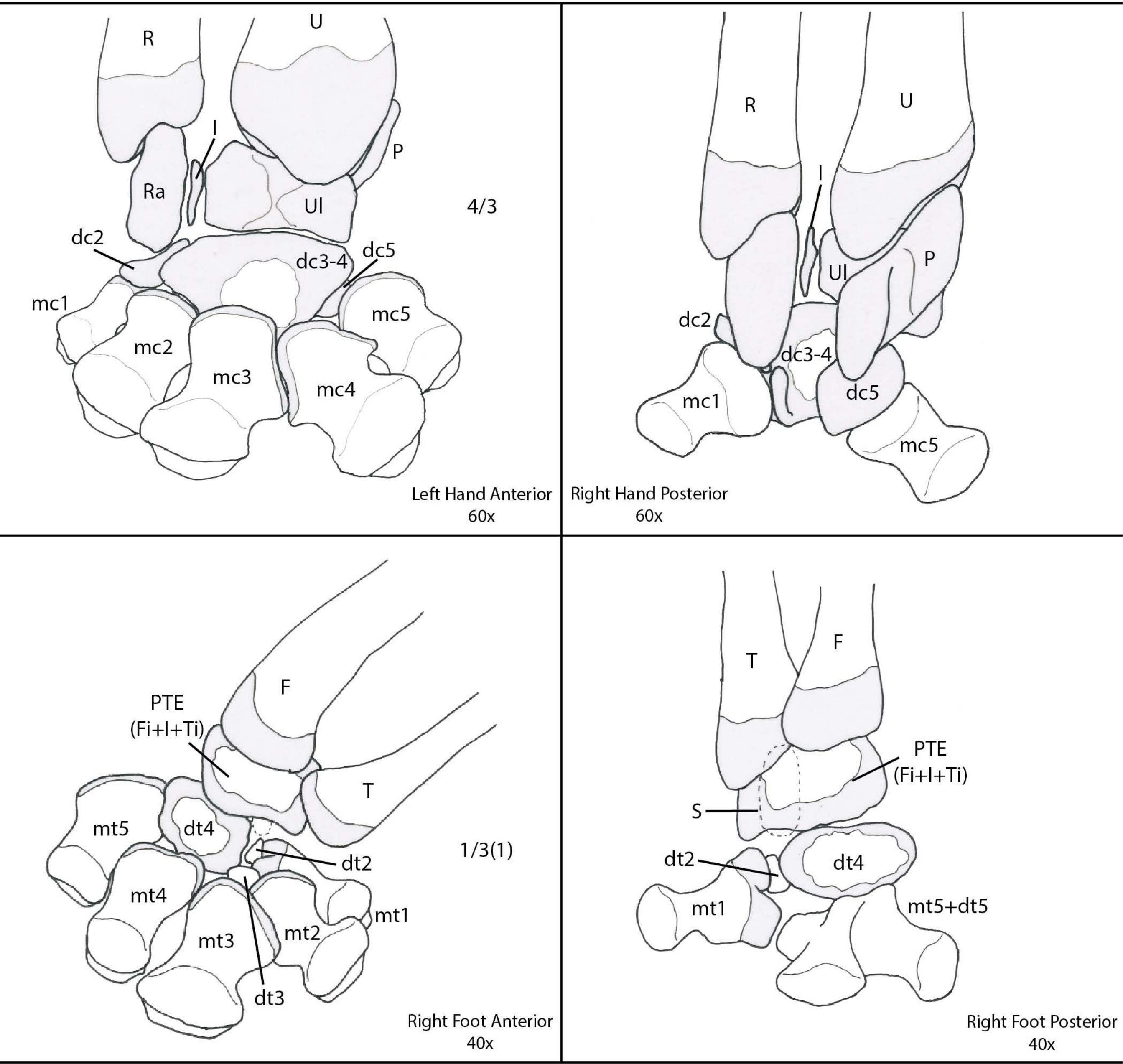

J) *Chamaeleo chameleon* CAS 132736

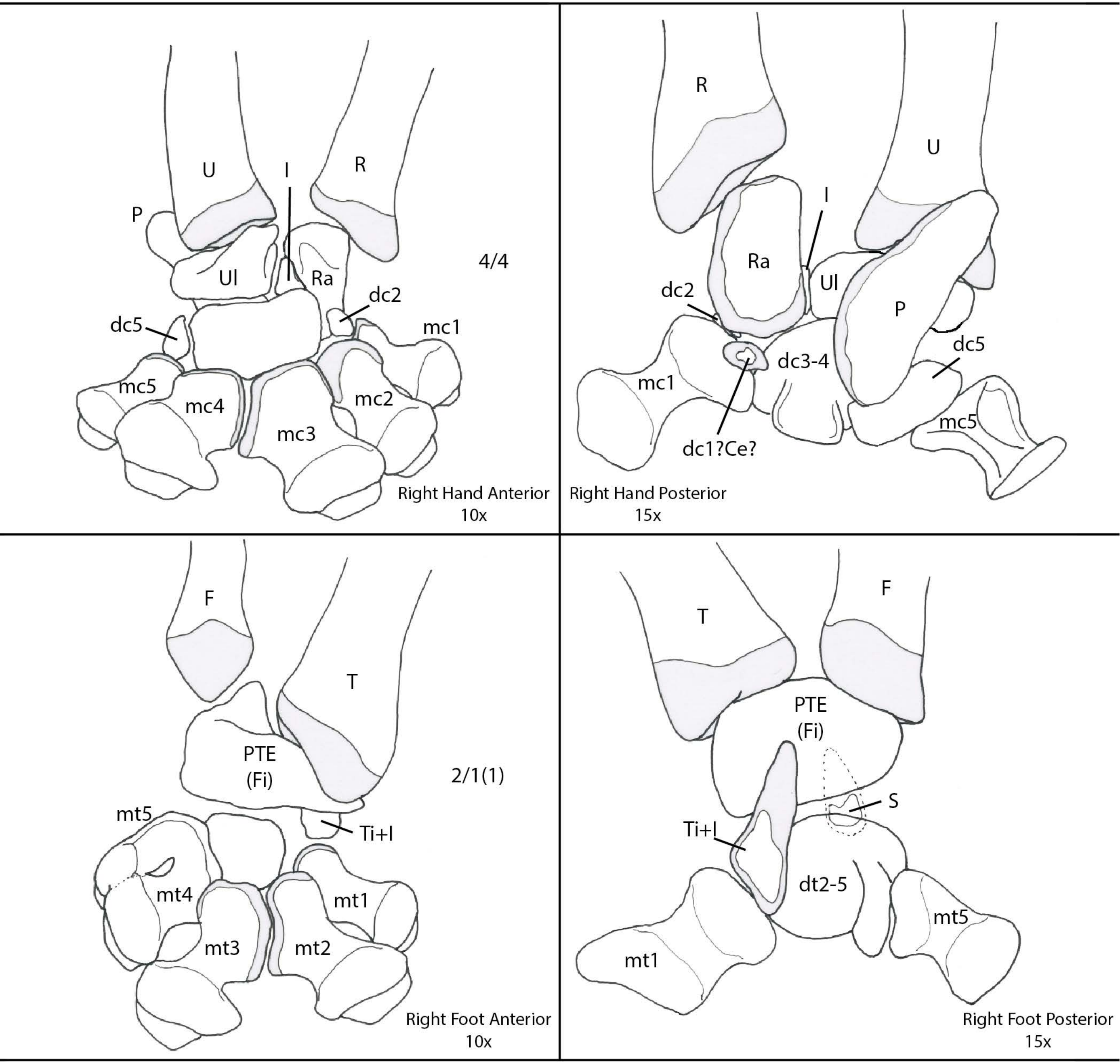

K) *Furcifer lateralis* CAS 123184

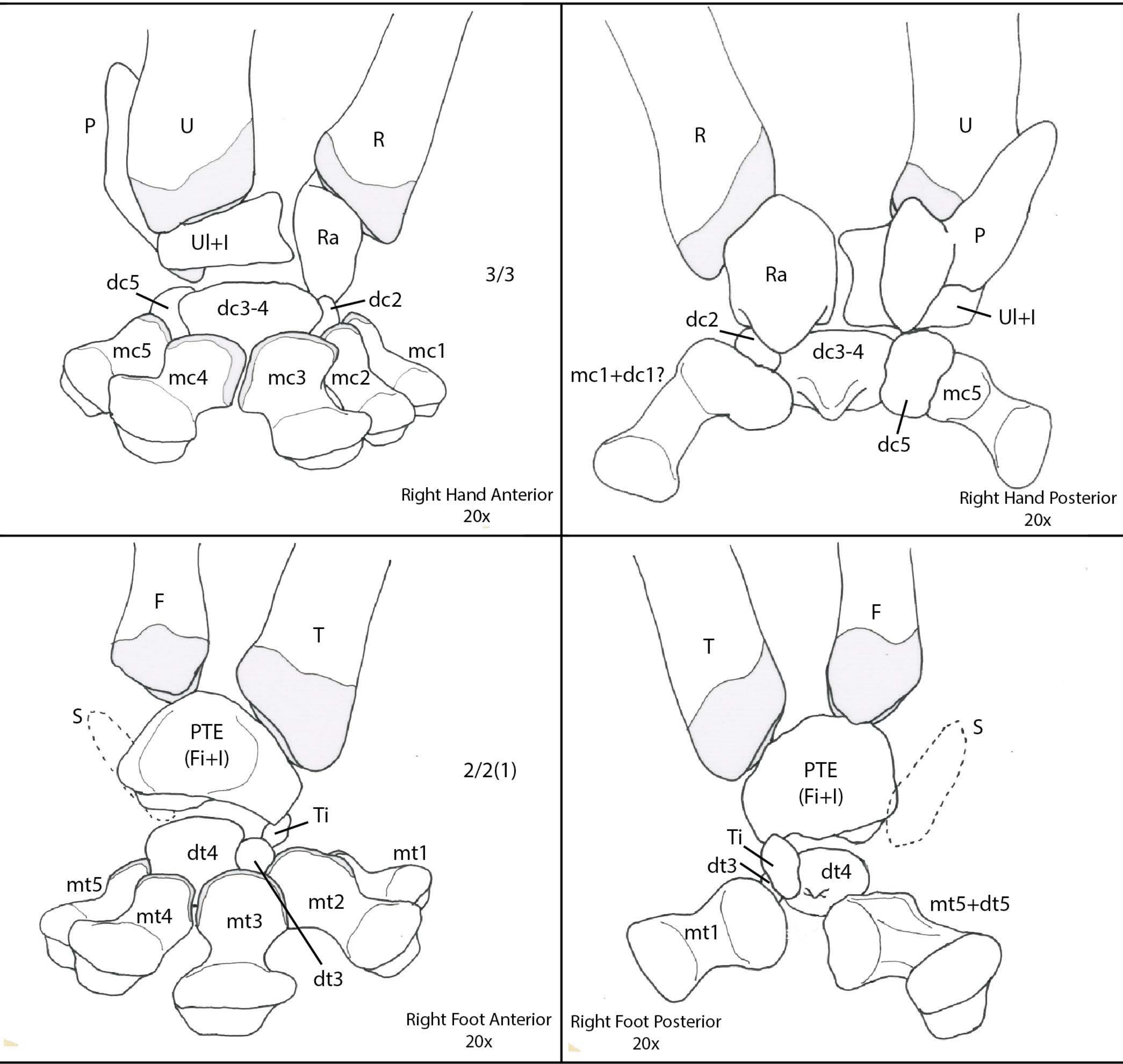

L) *Furcifer pardalis* CAS 156916

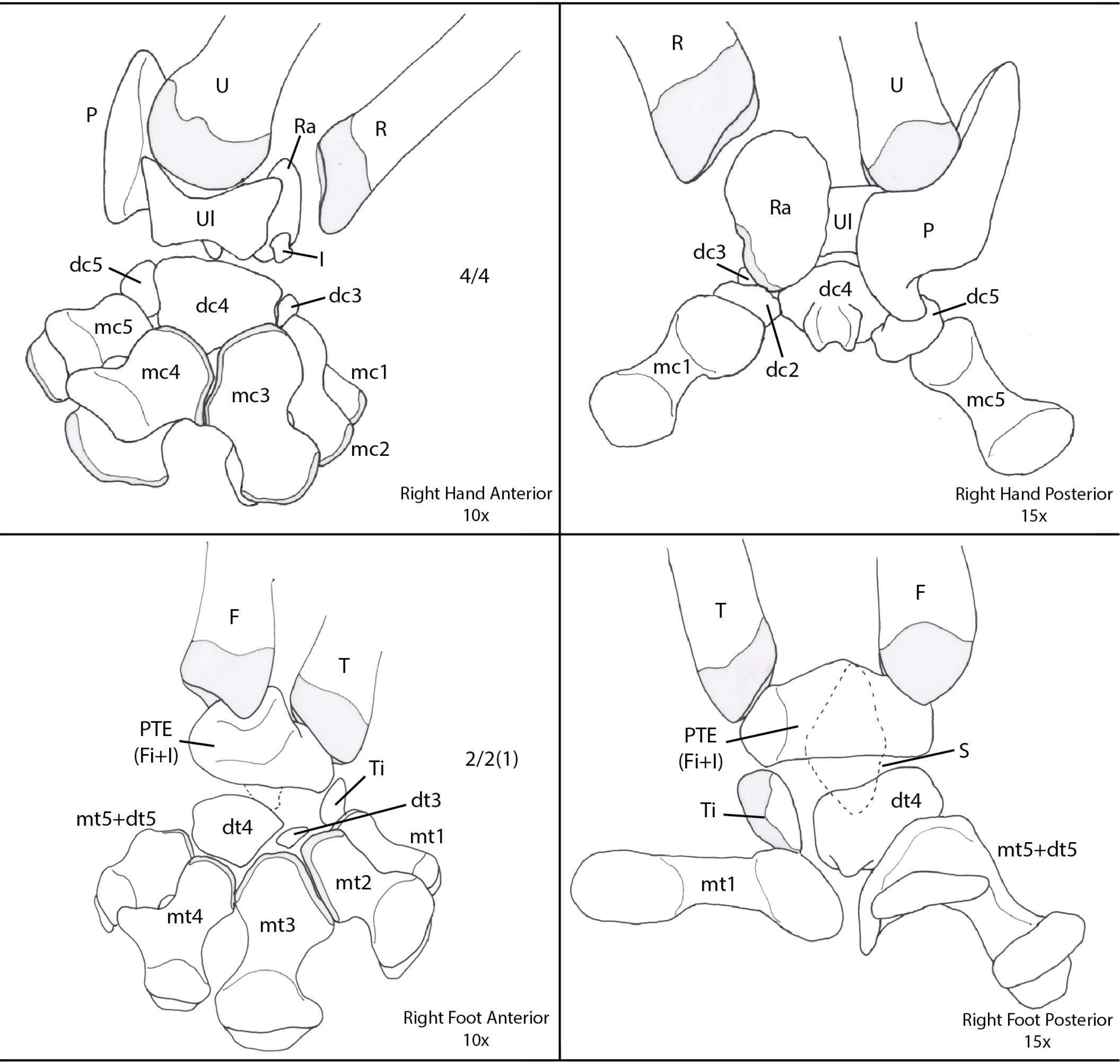

M) *Trioceros ellioti* CAS 201725

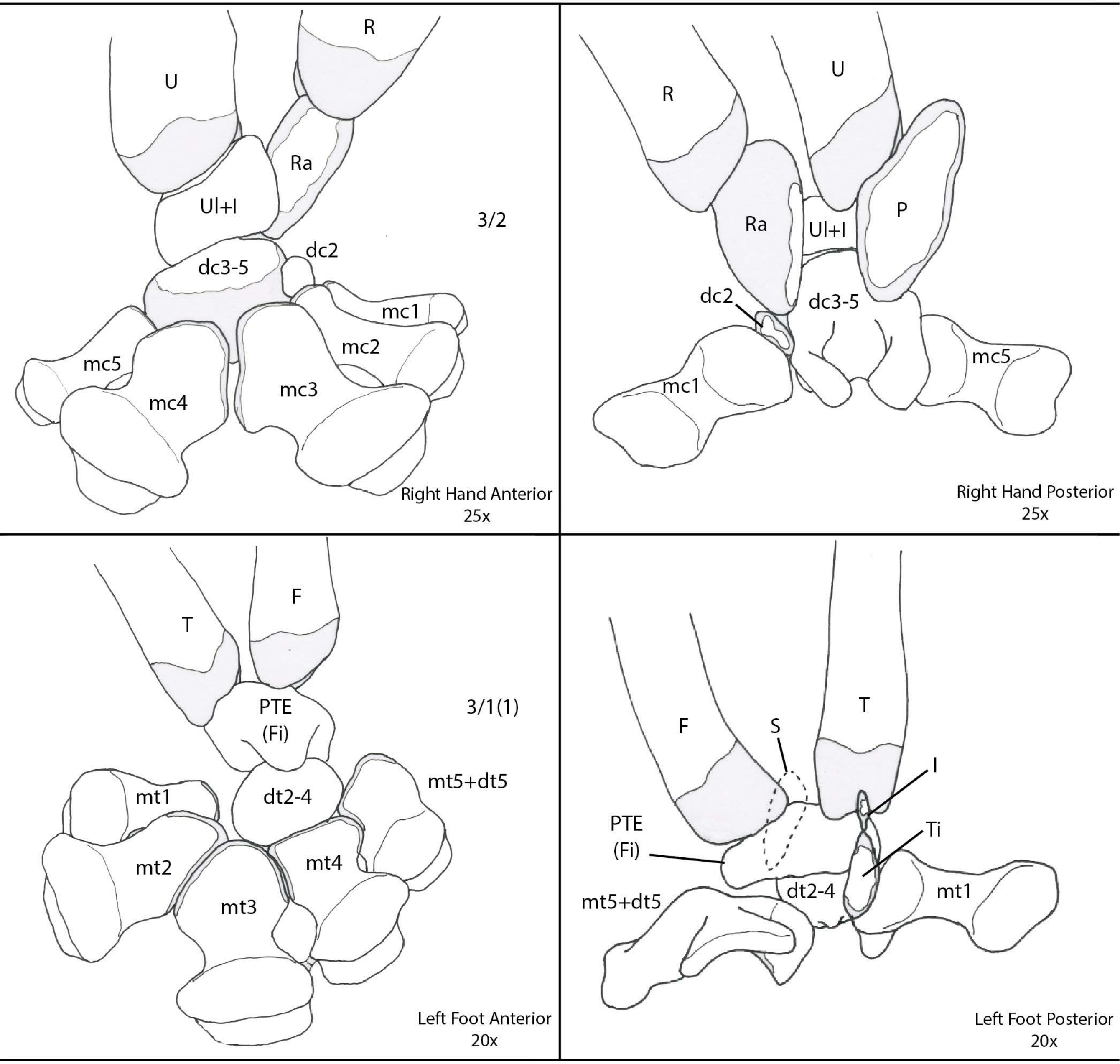

N) *Trioceros hohnelii* CAS 122181

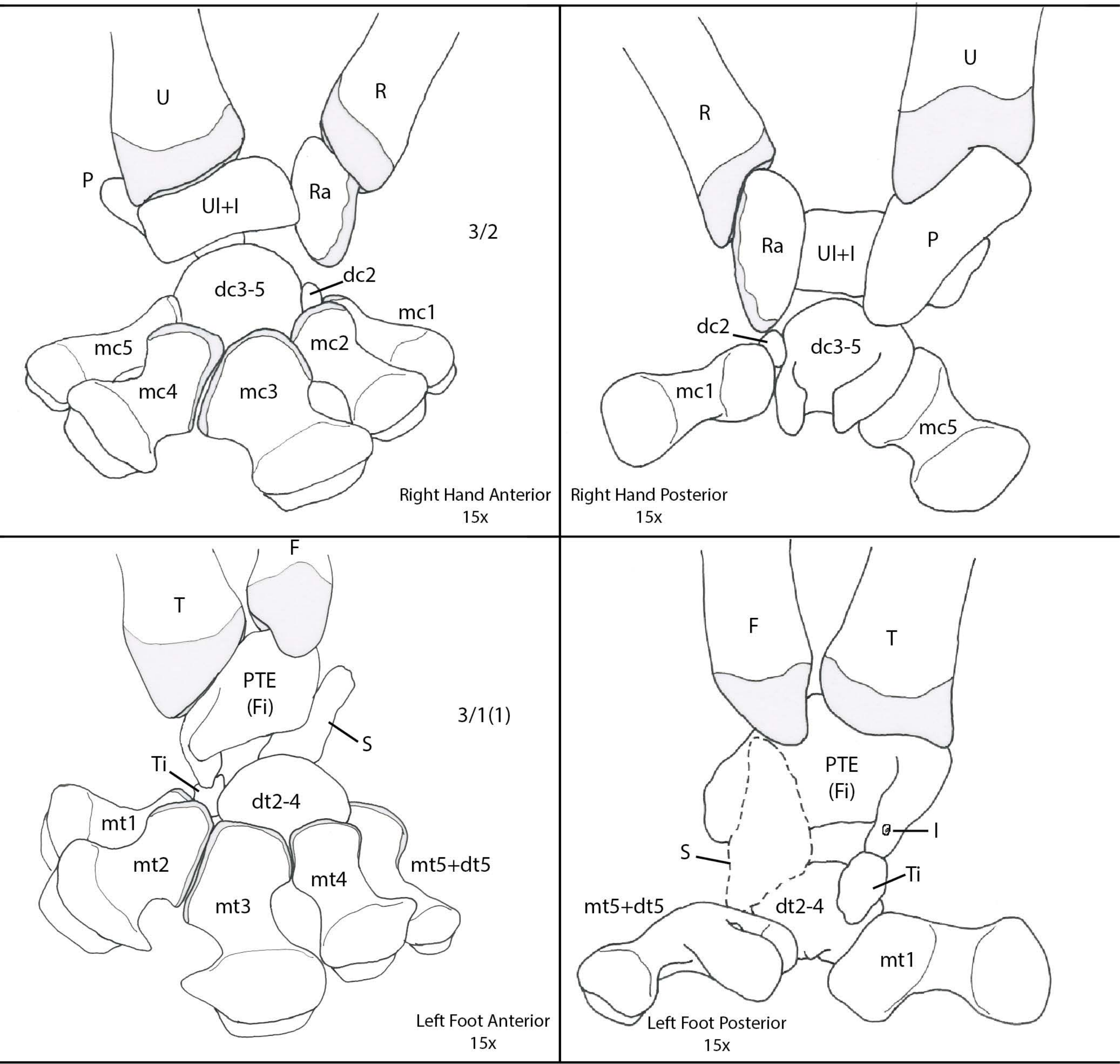

Supplement: Additional file 1: — Figure S1. Comparative morphology of autopodia in this study. Dorsoventrally flattened autopodia in outgroup taxa (Sphenodon punctatus, Aspidoscelis uniparens, Pogona vitticeps; A, B, C) were drawn in dorsal view only (with Sphenodon illustrations adapted from [72] and [94]) for both forelimb and hindlimb autopodia. D-N are chameleon illustrations from taxa cleared and stained in this study to look for ossified and chondrified elements comprising the autopodial architecture and are illustrated in both Anterior/Dorsal and Posterior/Ventral views. A) Sphenodon punctatus (Rhynchocephalia), B) Aspidoscelis uniparens (Teiidae), C) Pogona vitticeps (Agamidae), D) Brookesia stumpffi, E) Rieppeleon brevicaudatus, F) Rhampholeon boulengeri, G) Bradypodion pumilum, H) Chamaeleo dilepis, I) Chamaeleo calyptratus, J) Chamaeleo chameleon, K) Furcifer lateralis, L) Furcifer pardalis, M) Trioceros ellioti, N) Trioceros hohnelii. (PDF 2863 kb) [file 12862_2015_464_MOESM1_ESM.pdf]
