# Supplementary figures and images for: Hand/foot splitting and the ‘re-evolution’ of mesopodial skeletal elements during the evolution and radiation of chameleons
Source: BMC Evol Biol. 2015 Sep 18;15:184. doi: 10.1186/s12862-015-0464-4 (PMC4574539; doi:10.1186/s12862-015-0464-4)

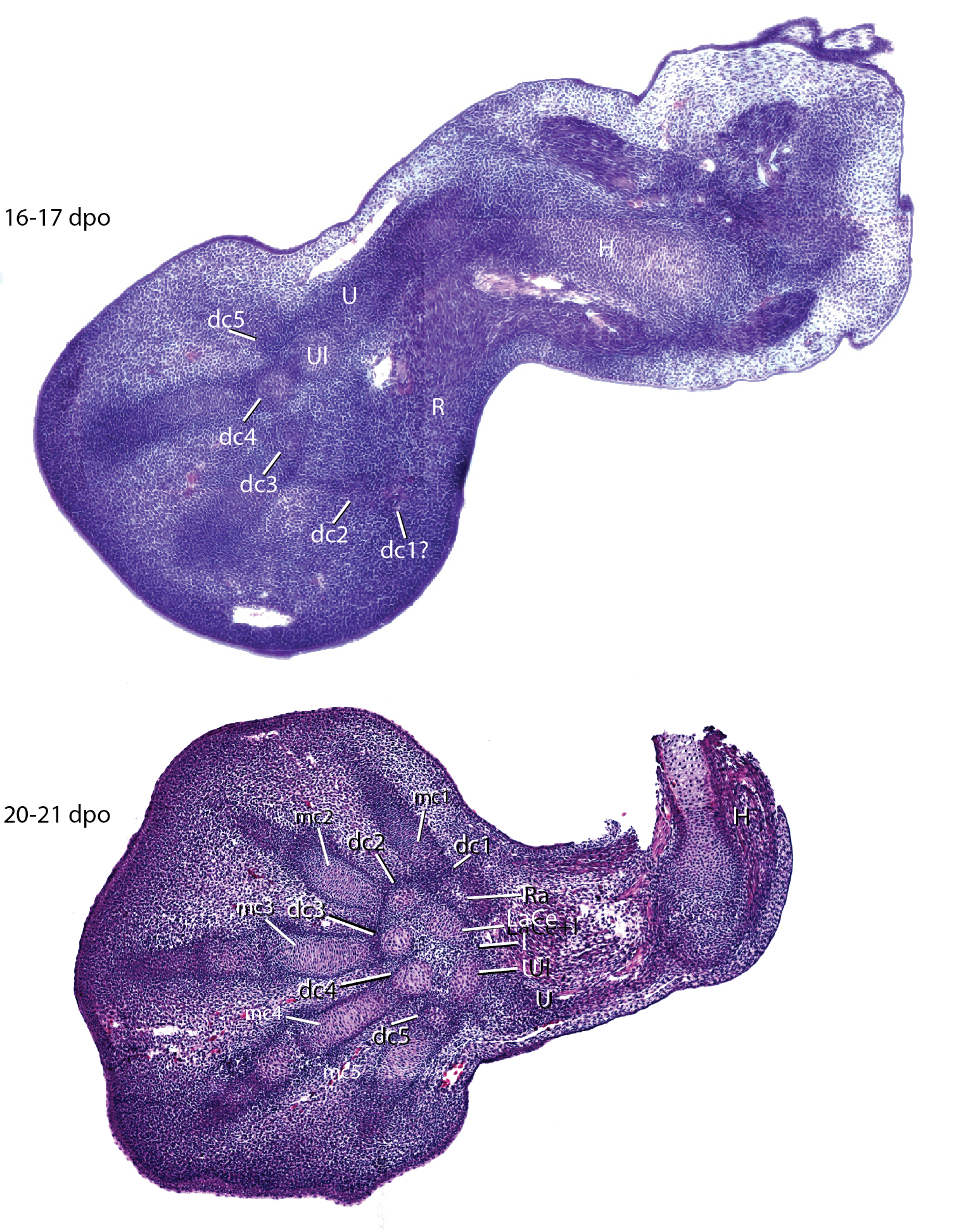

Supplement: Additional file 2: — Figure S2. Forelimb condensations of A. uniparens in H&E stained sections. A large contribution of preaxial mesenchymal cells contributing to digit 1/distal carpal 1 are visible in these sections as well as the development of an independent Lateral Centrale and Intermedium at an older stage. The Medial Centrale will later appear as a medial segmentation from the distal carpal 1. (TIFF 4374 kb) [file 12862_2015_464_MOESM2_ESM.tif]

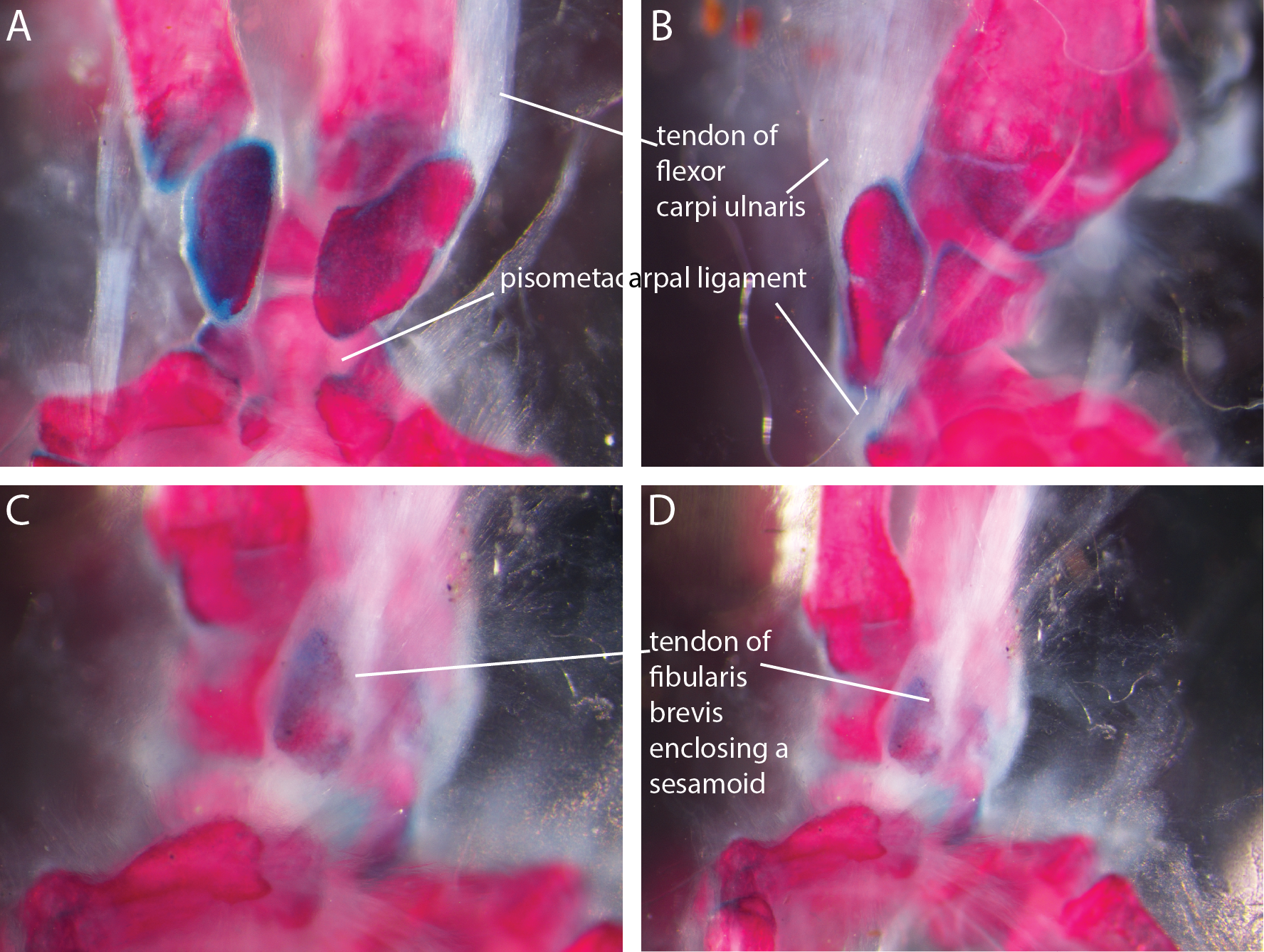

Supplement: Additional file 3: — Figure S3. Pisiform versus Sesamoid identity in the chameleon autopodium. The top row (A, B) shows the forelimb with the pisiform bone which we have identified in this study as segmenting from the ulnare. This bony element is not present within a tendon and instead has a proximal insertion of the tendon of the flexor carpi ulnaris with a more distal pisometacarpal ligament. The lower row (C, D) shows the hindlimb with a large sesamoid present within the tendon of the fibularis brevis which inserts onto the proximal margin of the ‘5th metatarsal.’ The species used for this image was Trioceros ellioti (CAS 201725). (TIFF 7809 kb) [file 12862_2015_464_MOESM3_ESM.tif]

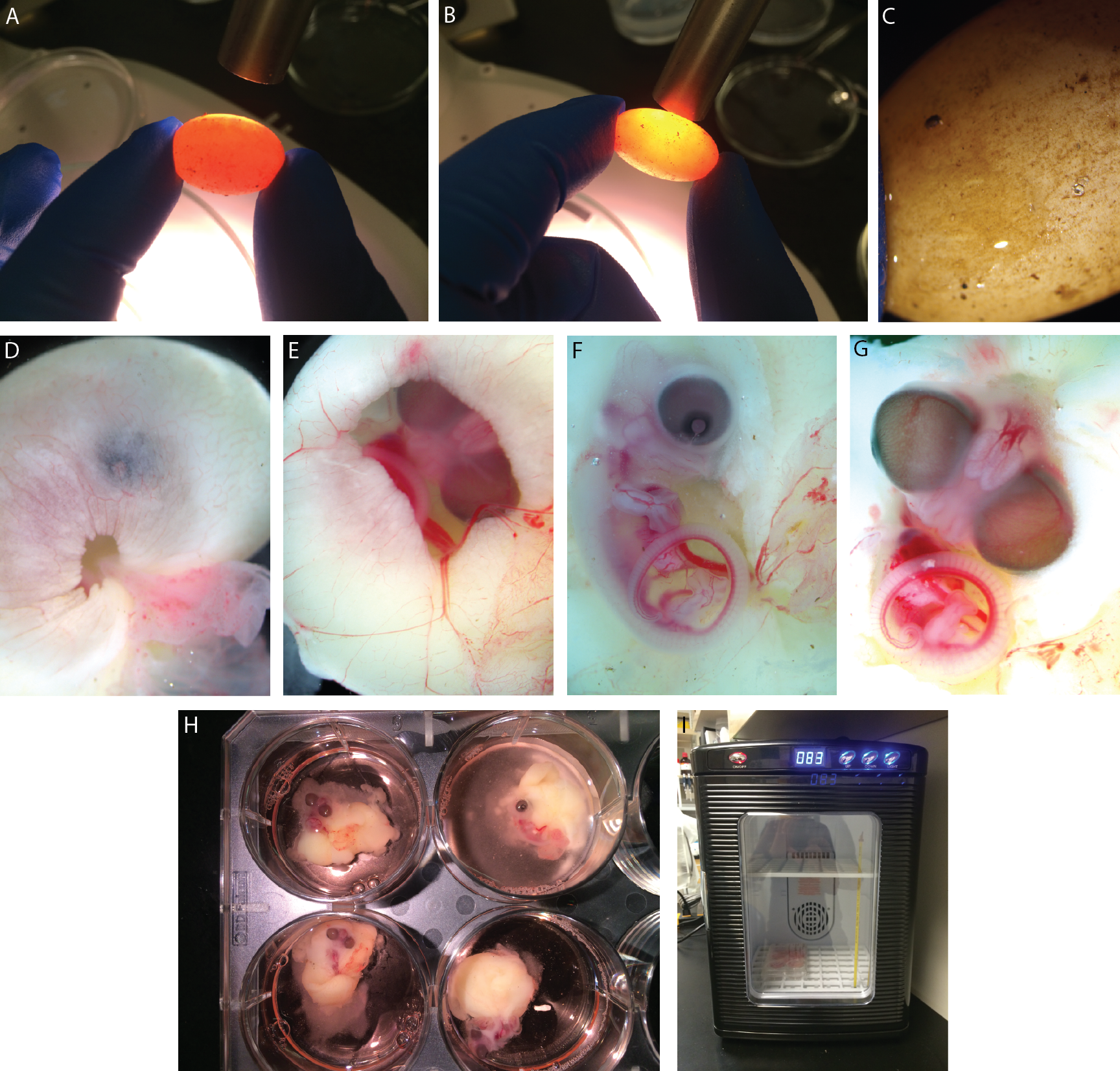

Supplement: Additional file 4: — Figure S4. Whole embryo culture of C. calyptratus. Details for this protocol are provided in the article text in the Methods section and correlate with labeled figures. (TIFF 7284 kb) [file 12862_2015_464_MOESM4_ESM.tif]

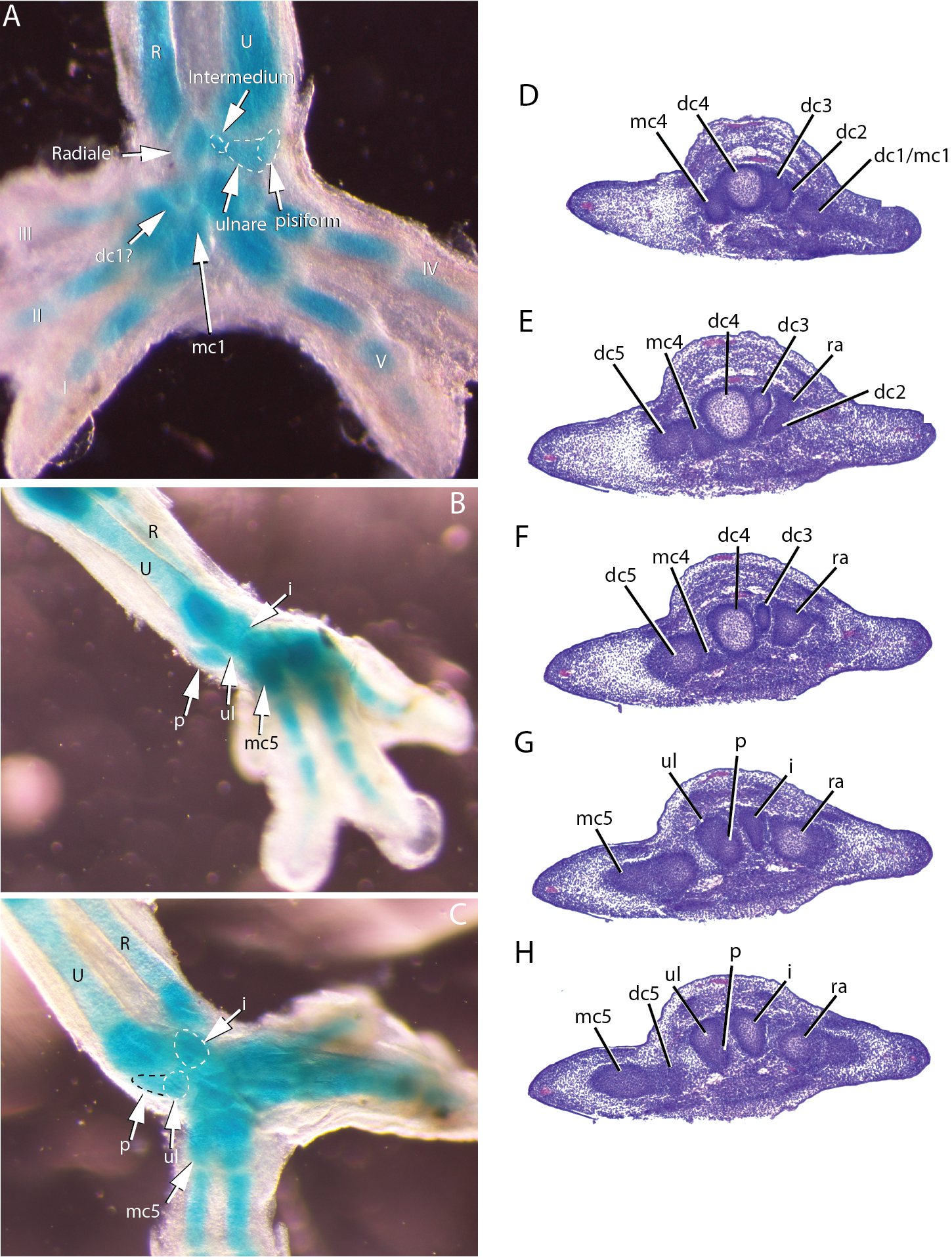

Supplement: Additional file 5: — Figure S5. Pisiform formation in the manus of C. calyptratus. Pisiform development from the ulnare is shown in posteroventral (A), lateral (B), and dorsal view (C) in whole alizarin/alcian skeletal preparations. Transverse H&E stained paraffin sections show, from distal to proximal mesopodium, the shared mesenchymal cells between the ulnare and the pisiform, supporting the ventrolateral segmentation of the pisiform from the ulnare (D-H). (TIFF 5828 kb) [file 12862_2015_464_MOESM5_ESM.tif]

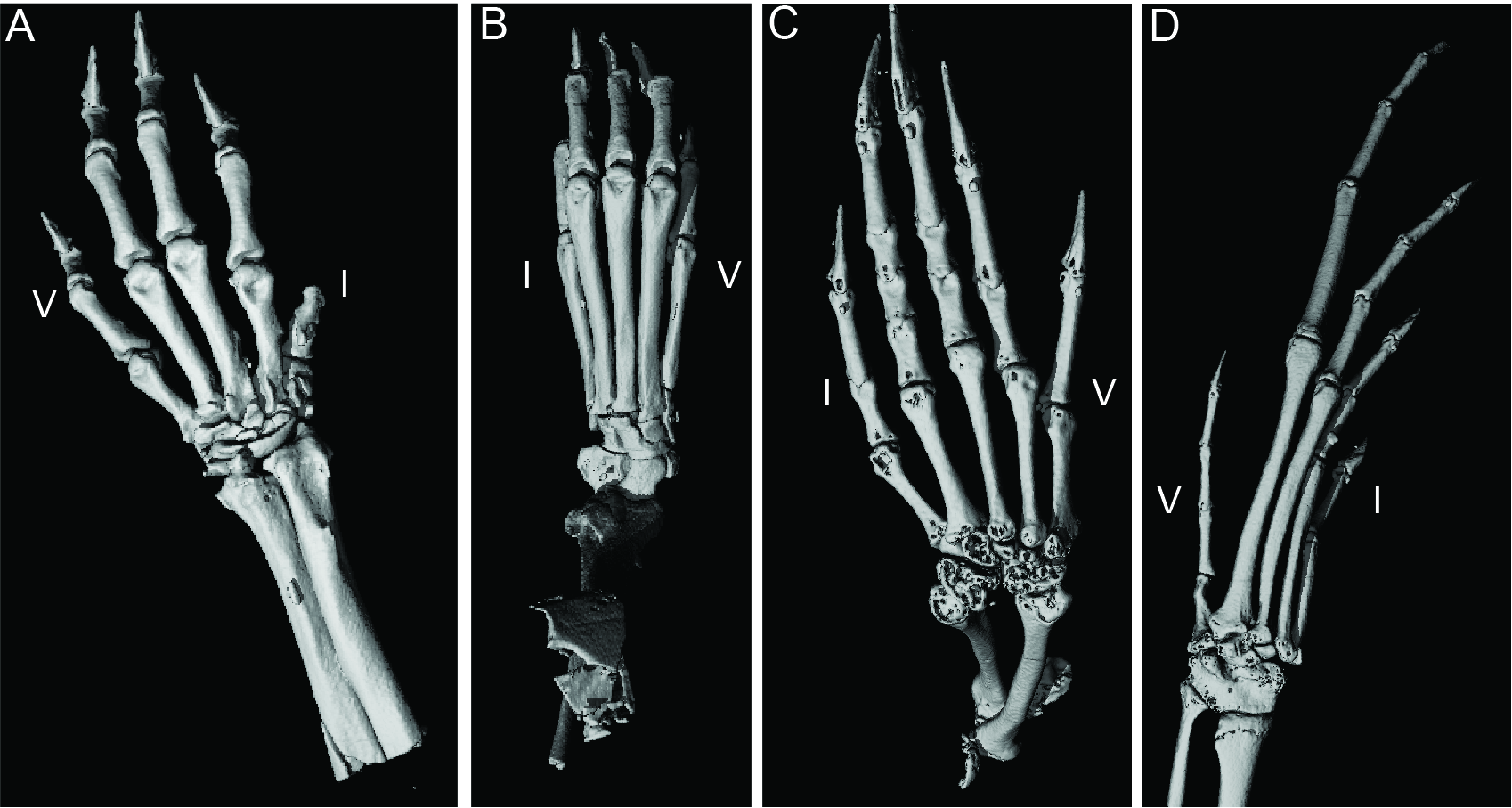

Supplement: Additional file 6: — Figure S6. Autopodia are dorsoventrally flattened Divergent tetrapods such as the common lab mouse (Mammalia) and the desert grassland whiptail lizard (Reptilia) have divergent hands (A, C) and feet (B, D). Despite architectural differences, they remain dorsoventrally flattened. (TIFF 1825 kb) [file 12862_2015_464_MOESM6_ESM.tif]
