# Supplementary material for: Hand/foot splitting and the ‘re-evolution’ of mesopodial skeletal elements during the evolution and radiation of chameleons
Source: BMC Evol Biol. 2015 Sep 18;15:184. doi: 10.1186/s12862-015-0464-4 (PMC4574539; doi:10.1186/s12862-015-0464-4)
Supplement: Additional file 7: — Chameleon specimen measurements. (DOCX 74 kb) [file 12862_2015_464_MOESM7_ESM.docx]

List of specimens used for illustrations with associated measurements and gender

CAS 167754 *Bradypodion pumilum*

SVL: 78.5 mm; TL: 65 mm

Male

CAS 156861 *Brookesia stumpffi*

SVL: 40.5 mm; TL: 33 mm

Male

CAS 132736 *Chamaeleo chamaeleon*

SVL: 141 mm; TL: 114 mm

Female; 14 ova, left oviduct: 16 ova, right oviduct

CAS 54687 *Chamaeleo dilepis*

SVL: 128 mm; TL: 133 mm

Female

CAS 201725 *Chamaeleo ellioti* (🡪 *Trioceros ellioti*)

SVL: 79.5 mm; TL: 68 mm

Female

CAS 122181 *Chamaeleo hohnelii* (🡪 *Trioceros hohnelii*)

SVL: 86 mm; TL: 65 mm

Female; 10 embryos right oviduct: 8 embryos left oviduct

CAS 123184 *Chamaeleo lateralis* (🡪 *Furcifer lateralis*)

SVL: 68.5; TL: 71 mm

CAS 156916 *Chamaeleo pardalis* (*Furcifer pardalis*)

SVL: 131 mm; TL: 133 mm

Female

CAS 176860 *Rhampholeon boulengeri*

SVL: 42.5 mm; TL: 10 mm

Male

*Rieppeleon brevicaudatus* (personal collection)

Adult; Female; 2 eggs

*Chamaeleo calyptratus* (personal collection)

Adult; Female

**Abbreviations**: SVL: Snout-Vent Length; TL: Tail Length; CAS: California Academy of Sciences
